# Cholesterol transfer proteins promote Atg-independent ER clearance by lysosomes

**DOI:** 10.1016/j.celrep.2026.117537

**Published:** 2026-06-08

**Authors:** Ruoxi Wang, Tina M. Fortier, Xiaofeng Sun, Fei Chai, Panagiotis D. Velentzas, Eric H. Baehrecke

**Affiliations:** 1 Department of Molecular, Cell and Cancer Biology, University of Massachusetts Chan Medical School, Worcester, MA 01605, USA; 2 Department of Neuroscience, School of Life Sciences, Southern University of Science and Technology, Shenzhen 518055, China; 3 Lead contact

## Abstract

Selective removal of endoplasmic reticulum (ER) is important for cell health. Macroautophagy is the primary mechanism for the removal of the ER, but the ER can be cleared in a macroautophagy-independent manner. However, the physiological relevance and mechanisms underlying macroautophagy-independent ER clearance remain largely unknown. Here we show that ER is cleared by lysosomes in a macroautophagy *Atg* gene-independent manner during development. This developmentally programmed *Atg*-independent ER clearance by lysosomes requires the ER protein Vap33 that promotes ER and lysosome contact. Oxysterol-binding protein (Osbp) is known to associate with Vap33, and Osbp lysosomal localization is required for ER clearance in cells lacking macroautophagy. Significantly, the cholesterol transport-associated protein Start1 regulates ER and lysosome contact, macroautophagy-independent ER clearance, and cholesterol transport from ER to the lysosome. These studies reveal that Vap33, Osbp, and Start1 promote ER clearance by lysosomes that is associated with cholesterol trafficking.

## In brief

The selective removal of endoplasmic reticulum (ER) is important for cell health. Wang et al. show that ER clearance occurs in a macroautophagy (*Atg*) gene-independent manner during development, and identify Vap33, Osbp, and Start1 as regulators of ER clearance by lysosomes that are associated with cholesterol trafficking.

## Graphical abstract

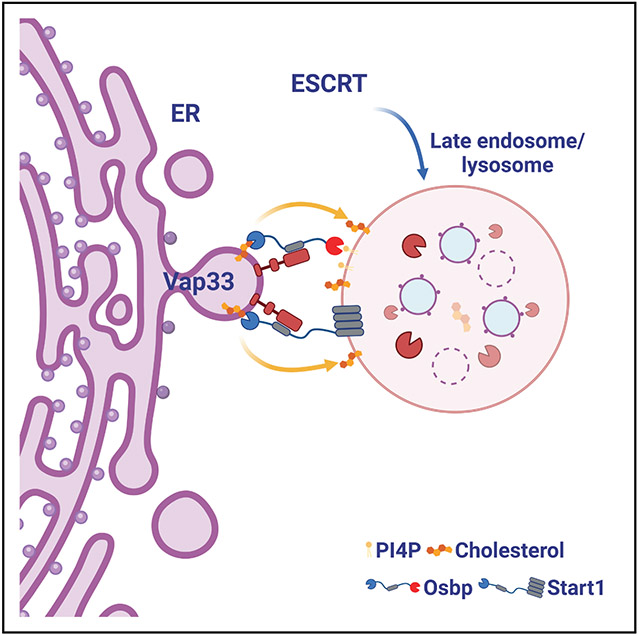

## INTRODUCTION

Autophagy is a catabolic process that degrades cytoplasmic materials by delivery to lysosomes, and alterations in autophagy have been associated with disease.^1^ Autophagy clears either bulk non-specific cytoplasmic materials or specific components, including the organelles endoplasmic reticulum (ER) and mitochondria.^2^ Macroautophagy is the most studied form of autophagy that is used for the selective removal of organelles. The selective removal of each type of organelle by macroautophagy depends on specific initiation signals and receptors.^3,4^ For example, the clearance of ER by macroautophagy (ER-phagy) depends on *Atg* genes and is conserved in diverse taxa.^5^ The ubiquitylation of ER-phagy receptors remodels the ER to facilitate the engulfment of intact ER cargoes into forming autophagosomes.^6-8^

Recent studies identified a process known as endosomal microautophagy that regulates the clearance of cytoplasmic components in yeast and cultured cells.^9-12^ During endosomal microautophagy, cytoplasmic cargoes are transported to the late endosome, and the endosomal sorting complexes required for transport (ESCRT) machinery facilitates the invagination of the endosome membrane for cytoplasmic cargo clearance.^11^ In contrast to ER-phagy, which is induced by either nutrient deprivation or during development, ER clearance by microautophagy is activated under chronic ER stress in cultured cells and yeast,^9,10^ raising questions about the relevance of macroautophagy-independent clearance of ER under physiological conditions in animals. Although microautophagy regulates chaperone-mediated reporter substrate clearance in *Drosophila* under starvation conditions,^13^ the mechanisms that control microautophagy are poorly understood. Thus, it remains unclear whether ER is removed by both macroautophagy gene-dependent and -independent mechanisms, and if these processes both contribute to ER clearance in a single cell under physiological conditions.

Here we show that ER clearance occurs in a macroautophagy *Atg* gene-independent manner during development as a normal part of animal physiology. We show that ER is cleared by both *Atg*-dependent and -independent mechanisms within the same *Drosophila* intestine enterocyte cell. Significantly, we discover that *Atg*-independent ER clearance is regulated by proteins known to influence the inter-organelle contact and transfer of cholesterol from ER to lysosomes. Our data indicate that Vap33, Osbp, and Start1 proteins promote the proximity of ER and lysosomes to enable ER clearance that is associated with cholesterol trafficking. These studies suggest that this mechanism regulates ER delivery to lysosomes based on cholesterol content.

## RESULTS

### The ESCRT functions in *Atg*-independent ER clearance during development

**Figure 1.**
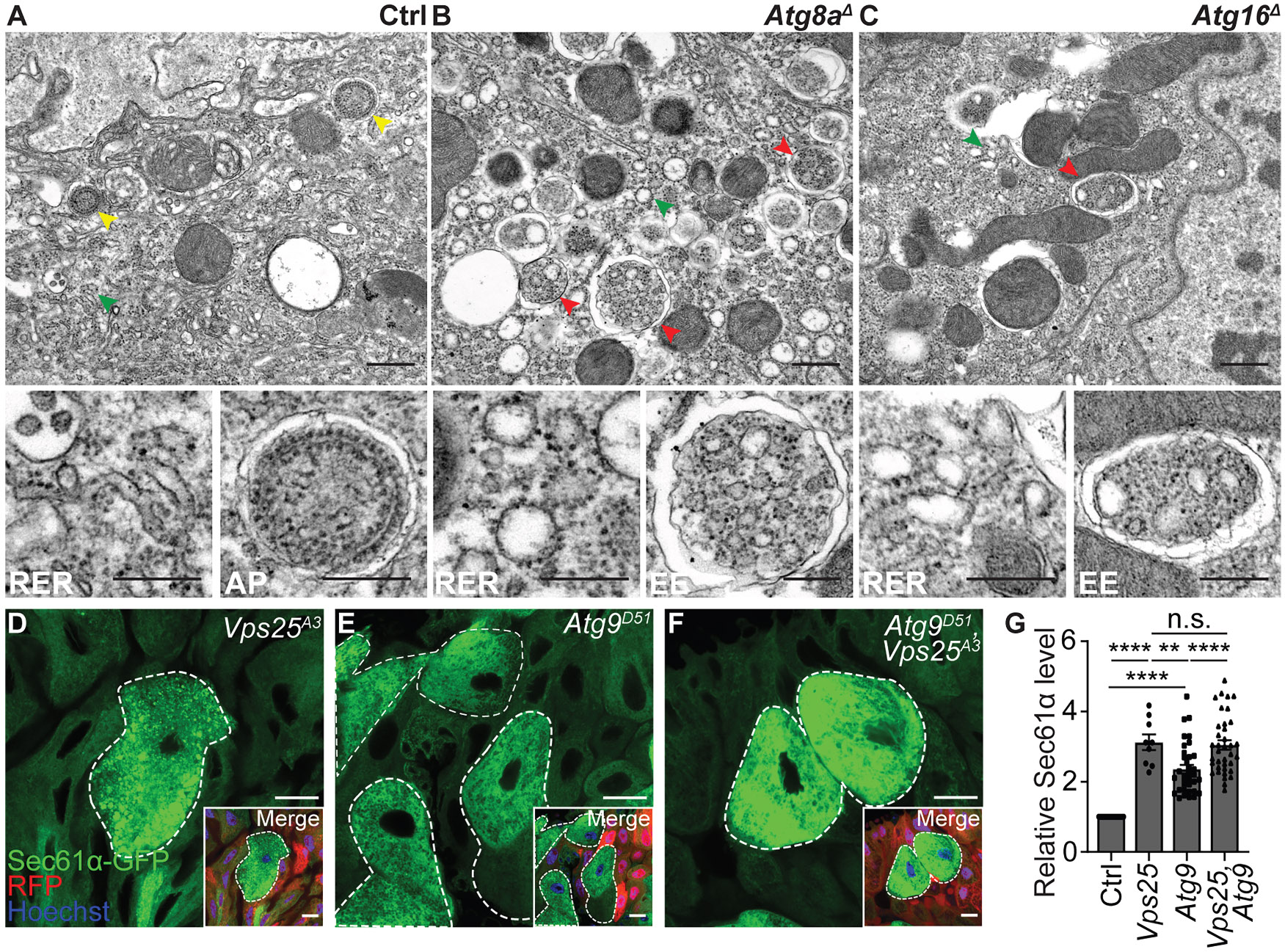
Lysosomal ER clearance requires both macroautophagy-dependent and -independent mechanisms (A–C) TEM images of homozygous *Atg8a*^*Δ*^ (B) and *Atg16*^*Δ*^ (C) mutant enterocytes exhibit increased small dilated rough ER surrounded by ribosomes both inside and outside of endosomes/lysosomes compared to control *w*^*1118*^ (A), which possesses large intact rough ER in autophagosomes. Yellow arrows in (A) indicate ER in an autophagosome (AP), and green arrow indicates an autolysosome (ALY). Red arrows in (B) and (C) represent early endosomes (EE), and blue arrows indicate endolysosomes (EL) containing rough ER. (D–F) *Vps25*^*A3*^ and *Atg9*^*D51*^ double mutant enterocyte cells (F, white dotted line, non-red) possess similar levels of Sec61α-GFP (green) compared to *Vps25*^*A3*^ mutant enterocytes (D, white dotted line, non-red), and increased Sec61α levels compared to *Atg9*^*D51*^ mutant enterocytes (E, white dotted line, non-red). (G) Quantification of Sec61α-GFP intensity in each genotype, normalized to neighboring control cells. *n* = 81 (Ctrl), *n* = 9 (*Vps25*), *n* = 35 (*Atg9*), *n* = 37 (*Vps25*, *Atg9*) cells were measured. Animals were staged 2 h APF. (D-F) mutant cells lacking RFP (red) are indicated by white dotted lines. Scale bars in (A-C) represent 0.5 μm, scale bars in (A-C) insets represent 0.25 μm; Scale bars in (D-F) and insets represent 20 μm. Data are presented as mean ± SEM. n.s. = not significant, **p < 0.01, ****p < 0.0001 from one-way ANOVA corrected by Tukey’s post hoc test and unpaired, two-tailed *t* test. Each data point represents one mutant cell/neighboring cell. Representative of 3 or more independent biological experiments from ≥3 different animals.

The selective removal of ER by delivery to the lysosome can be triggered by multiple types of cell stress, and was also recently reported to occur as part of a normal developmental program in *Drosophila*.^8^ Transmission electron microscopy (TEM) analysis of control *Drosophila* intestine enterocyte cells revealed intact rough ER with ribosomes in double membrane autophagosomes 2 h after puparium formation (APF) (Figure 1A). By contrast, *Atg8a*^*Δ*^ mutant enterocytes that lack macroautophagy^8^ exhibit increased amounts of small, dilated rough ER either in the cytoplasm or in late endosomes/lysosomes 2 h APF (Figure 1B). Consistent with these results, analysis of a different core macroautophagy *Atg16*^*Δ*^ gene mutant enterocytes lacking macroautophagy^8,14^ possess similar rough ER structures in endosomes/lysosomes (Figure 1C), suggesting that ER is delivered to late endosomes/lysosomes in intestine cells lacking macroautophagy during development.

ESCRT-dependent regulation of cargo invagination into late endosomes is a key step during endosomal microautophagy.^11,15,16^ Therefore, we next tested if the ESCRT is required for ER clearance in developing intestinal cells. Consistent with this hypothesis, *Vps25* (ESCRT-II) RNAi knockdown enterocytes possess increased dilated rough ER structures and fail to deliver the ER to either endosome or lysosome structures compared to control cells expressing *Luciferase* RNAi (Figures S1A-S1C). In addition, *Vps25*^*A3*^ loss-of-function mutant enterocytes, which lack red fluorescent protein (RFP), exhibit increased intensity of the ER marker Sec61α-GFP compared to *Atg9*^*D51*^ single mutant cells 2 h APF (Figures 1D, 1E, and 1G). *Atg9*^*D51*^ mutants were used to facilitate double mutant analysis because *Atg9* is on the same chromosome arm as *Vps25*. Since *Atg8a* and *Atg9* act at distinct stages in macroautophagy, we first tested if these mutations have a similar impact on macroautophagy-dependent cargo clearance. *Atg8a*^*Δ*^*Atg9*^*D51*^ double mutant enterocytes possess similar levels of mitochondrial ATP5A antibody-stained puncta compared to either of *Atg8a*^*Δ*^ or *Atg9*^*D51*^ single mutant cells that were all induced in the same intestines (Figures S1D– S1F). Importantly, *Vps25*^*A3*^ and *Atg9*^*D51*^ double mutant cells possess similar levels of Sec61α-GFP compared to *Vps25*^*A3*^ single mutant cells, and increased Sec61α-GFP intensity compared to *Atg9*^*D51*^ single mutant cells (Figures 1D-1G). These data suggest that the ESCRT regulates cargo degradation by both macroautophagy and *Atg*-independent clearance of ER within the same cell.

To further investigate if ER is delivered to lysosomes in the absence of macroautophagy, we utilized an established ER-localized ss-pHluorin-mKate2-KDEL-V5 sensor (Figure S1G)^8^ to measure ER clearance by lysosomes. The acidic environment of the lysosome suppresses green fluorescence of pHluorin but retains red fluorescence of mKate2 when the ER containing the sensor is delivered to the lysosome. Since developmentally programmed macroautophagy regulates *Drosophila* enterocyte cell size reduction,^8,17^ we identified enterocytes that lack macroautophagy based on their larger cell size compared to neighboring smaller macroautophagy-competent control cells. Larger *Lsn*^*SS6*^ (Vps22, ESCRT-II) loss-of-function mutant cells exhibit complete co-localization of ss-pHluorin-KDEL and ss-mKate2-KDEL puncta and decreased LysoTracker puncta compared to smaller neighboring control cells (Figures S1H and S1K). In addition, ss-pHluorin-KDEL and LysoTracker co-localization is decreased in *Lsn* and *Atg16* double mutant enterocytes compared to *Atg16* single mutant enterocytes 2 h APF (Figures S1H-S1K). These data indicate that the ESCRT is required for ER clearance by delivery to lysosomes that is independent of *Atg* gene function.

### ER and lysosome contact regulates *Atg*-independent ER clearance

**Figure 2.**
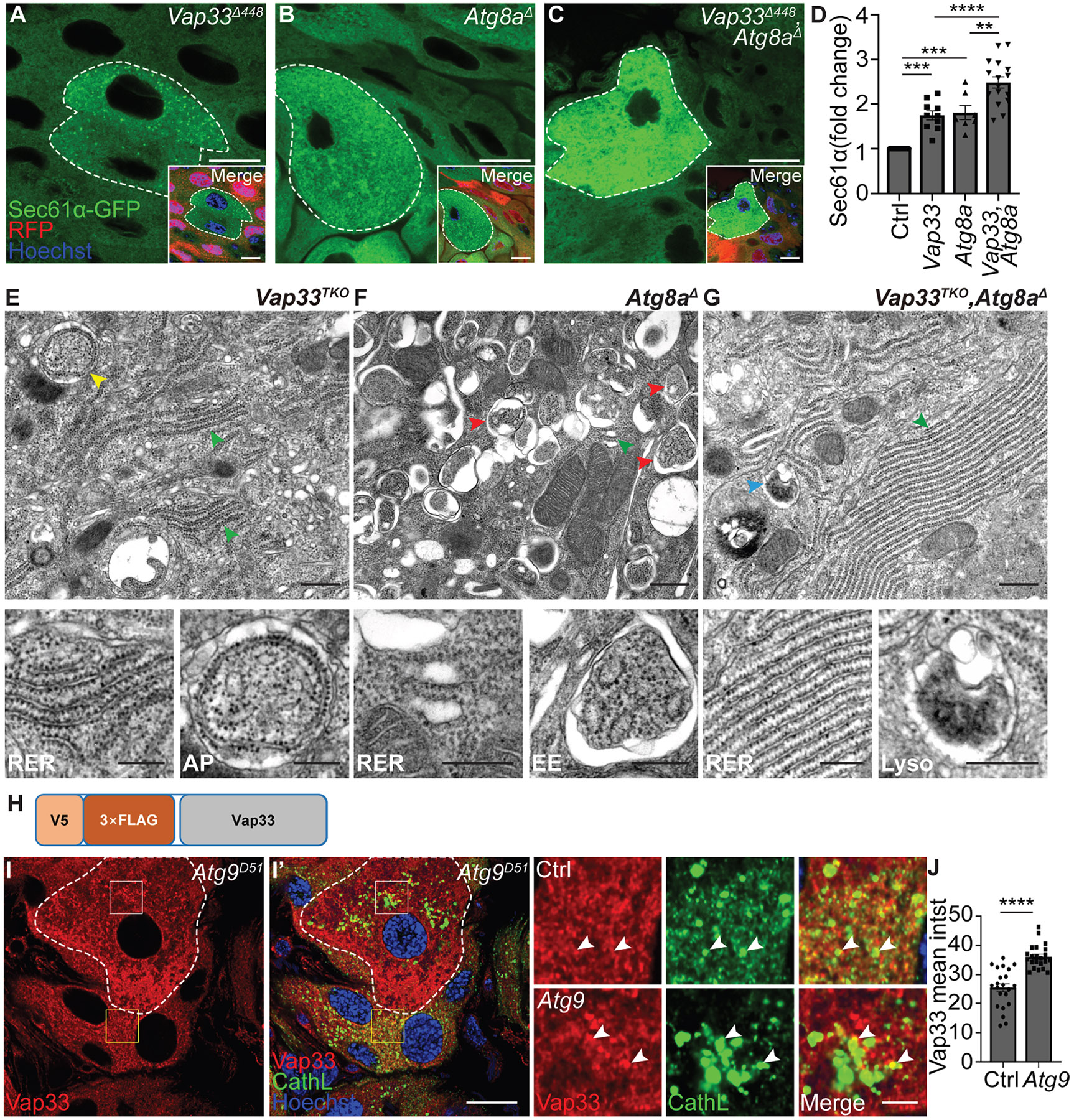
Vap33 regulates ER clearance independent of macroautophagy (A–C) *Vap33*^*Δ448*^ *Atg8a*^*Δ*^ double mutant enterocyte cells (C) possess enhanced Sec61α-GFP levels (green) compared to either *Vap33*^*Δ448*^ (A) or *Atg8a*^*Δ*^ (B) single mutant cells. (D) Quantification of Sec61α-GFP intensity in each genotype normalized to neighboring control cells. *n* = 33 (Ctrl), *n* = 10 (*Vap33*), *n* = 7 (*Atg8a*), and *n* = 16 (*Vap33*, *Atg8a*) cells were measured. (E) TEM images of *Vap33*^*TKO*^ single mutant cells that exhibit normal ER sheets and ER structures in autophagosomes. (F) TEM images of *Atg8a*^*Δ*^ single mutant cells exhibit dilated rough ER either in the cytoplasm or in endosome-like structures. (G) TEM images of *Vap33*^*TKO*^ *Atg8a*^*Δ*^ double mutant cells exhibit increased rough ER parallel sheets in the cytoplasm compared to either *Vap33* or *Atg8* single mutant cells. (H) Schematic of V5–3×FLAG-Vap33. (I and I′) Larger *Atg9*^*D51*^ mutant enterocytes (white dotted line) exhibit increased V5–3×FLAG-Vap33 (Vap33, red) intensity and puncta co-localization with Cathepsin L puncta (green) compared to smaller neighboring control cells. (J) Quantification of Vap33 intensity in *Atg9* mutant enterocytes compared to neighboring control cells. *n* = 22 (Ctrl) and *n* = 22 (*Atg9*) cells were measured. Animals were staged 2 h APF. (A–C) mutant cells lacking RFP (red) are indicated by white dotted lines. Scale bars in (E–G) represent 0.5 μm, and in (E-G) insets represent 0.25 μm; Scale bars in (A–C) and insets (I′) represent 20 μm, and (I) insets represent 5 μm. Insets in (I) are from indicated rectangles (white rectangle = mutant cell, yellow rectangle = control cell). White arrows in (I) indicate co-localization of Vap33 and Cathepsin L. Data are presented as mean ± SEM. **p < 0.01, ***p < 0.001, and ****p < 0.0001 from one-way ANOVA corrected by Tukey’s post hoc test and unpaired, two-tailed *t* test. Each data point represents one mutant cell/ neighboring cell. Representative of 3 or more independent biological experiments from ≥3 different animals.

We next sought to identify genes that have not been previously implicated in *Atg*-independent clearance of ER. ER-localized VAMP-associated protein A or B (Vap-A or Vap-B) interacts with proteins to facilitate ER contact with either late endosomes or lysosomes.^18,19^ Therefore, we tested whether the *Drosophila* Vap-A/B orthologue Vap33 is required for ER clearance. Interestingly, *Vap33*^*Δ448*^ loss-of-function mutant enterocytes that lack RFP possess elevated intensity of Sec61α-GFP compared to neighboring control RFP-positive cells 2 h APF (Figures 2A and 2D). Importantly, *Vap33* and *Atg8a* double mutant RFP-negative enterocytes possess increased Sec61α intensity compared to either *Vap33* or *Atg8a* single mutant enterocytes 2 h APF (Figures 2A-2D). Consistent with these data, *Vap33* and *Atg8a* double mutant enterocytes possess less ss-mKate2-KDEL puncta co-localization with LysoTracker compared to *Atg8a* single mutant enterocytes 2 h APF (Figures S2A-S2C). Importantly, *Vap33* mutant enterocytes possess similar lysosomal enzyme Cathepsin L puncta compared to neighboring control cells (Figures S2D and S2G), and *Vap33 Atg8a* double mutant enterocytes exhibit similar amounts of Cathepsin L puncta compared to *Atg8a* single mutant cells (Figures S2D-S2G). In addition, *Vap33* mutant enterocytes possess similar mitochondrial ATP5A, autophagic cargo receptor Ref(2)p, and Atg8a puncta compared to control cells, suggesting that Vap33 is not required for macroautophagy (Figures S2H-S2L), since these proteins accumulate in *Atg* mutants.^8,14,17^ Significantly, TEM analysis revealed a striking difference between *Atg8a*^*Δ*^*Vap33*^*TKO*^ double mutant enterocytes that fail to exhibit ER in degradative structures compared to either *Vap33* or *Atg8a* single mutant control cells (Figures 2E-2G). In addition, the morphology of long parallel sheets of rough ER in *Atg8a*^*Δ*^*Vap33*^*TKO*^ double mutant enterocytes is very different from *Atg8a*^*Δ*^ single mutant cells that possess small dilated rough ER either in the cytoplasm or in late endosomes/lysosomes 2 h APF (Figures 2F and 2G). Combined, these data indicate that Vap33 functions in a parallel pathway with Atg8a to clear the ER.

We next investigated whether macroautophagy influences Vap33 levels. Since antibodies against Vap33 do not exist, we used CRISPR-Cas9 gene editing to tag the N terminus of Vap33 with 3×FLAG-V5 to enable the detection of all Vap33 isoforms (Figure 2H). Loss of *Atg9* resulted in elevated levels of Vap33 compared to control enterocytes 2 h APF (Figures 2I and 2J). In addition, Vap33 forms larger puncta that are co-localized with the lysosome enzyme Cathepsin L in *Atg9* mutant compared to control cells (Figure 2I). These data suggest a complementary relationship between macroautophagy and Vap33-dependent ER clearance.

**Figure 3.**
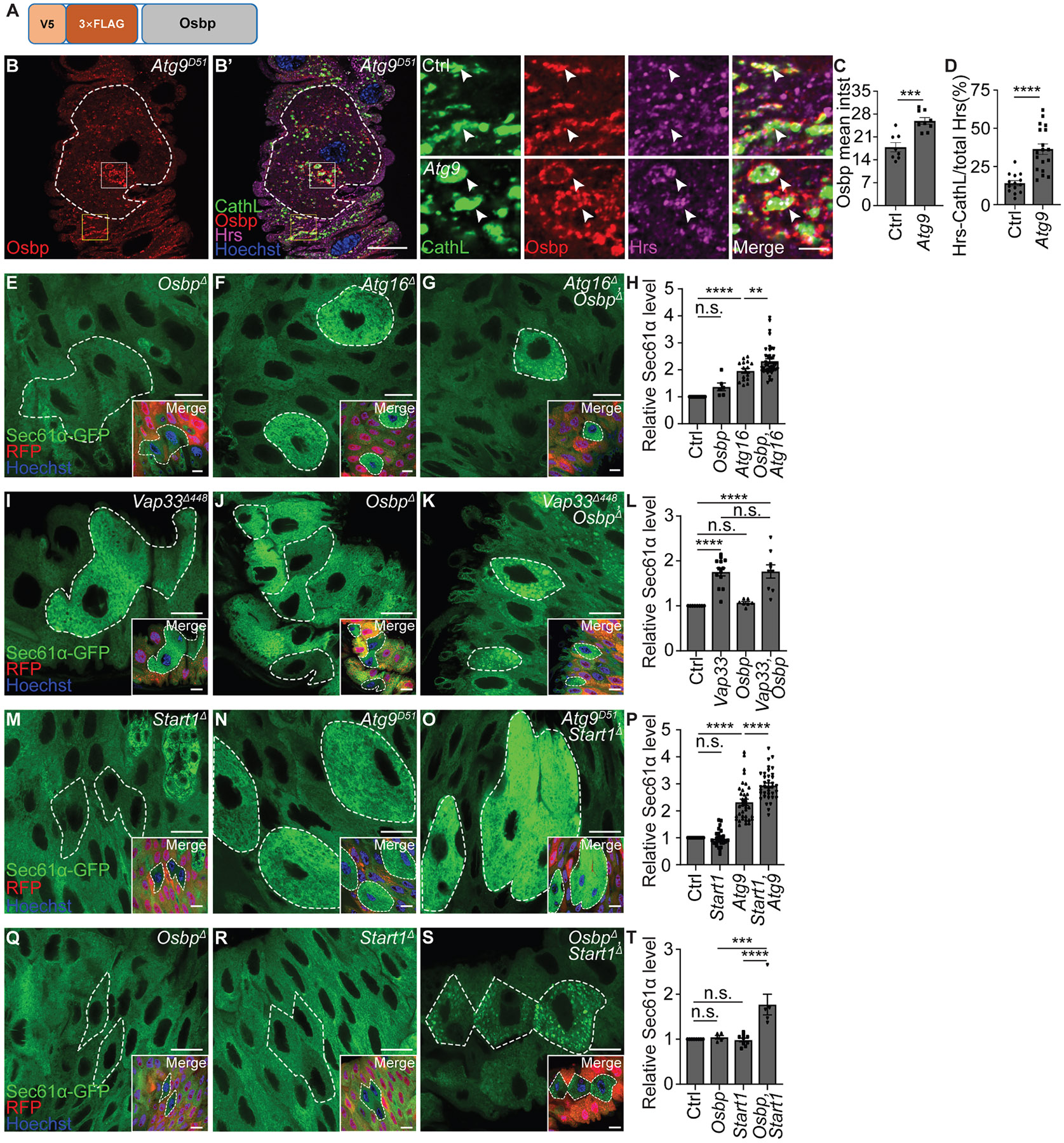
Osbp and Start1 are required for *Atg*-independent lysosomal ER clearance (A) Schematic of V5–3×FLAG tagged Osbp. (B and B′) *Atg9*^*D51*^ mutant enterocyte cells (white dotted line) exhibit increased V5–3×FLAG-Osbp (Osbp, red) and Hrs (magenta) puncta surrounding Cathepsin L puncta (Cath L, green) compared to smaller neighboring control cells. (C) Quantification of Osbp mean intensity in *Atg9* mutant enterocytes compared to neighboring control cells. *n* = 8 (Ctrl) and *n* = 9 (*Atg9*) cells were measured. (D) Quantification of the ratio of co-localized Hrs and Cathepsin L puncta of total Cathepsin L puncta in *Atg9* mutant enterocytes compared to control cells. *n* = 14 (Ctrl) and *n* = 18 (*Atg9*) cells were measured.. (E–G) *Osbp*^*Δ*^ *Atg16*^*Δ*^ double mutant enterocyte cells (G) possess increased levels of Sec61α-GFP (green) compared to either *Osbp*^*Δ*^ (E) or *Atg16*^*Δ*^ (F) single mutant cells. *Osbp*^*Δ*^ single mutant cells possess similar Sec61α-GFP levels as neighboring control cells. (H) Quantification of Sec61α-GFP intensity in enterocytes of each mutant genotype normalized to neighboring control cells. *n* = 64 (Ctrl), *n* = 6 (*Osbp*), *n* = 16 (*Atg16*), and *n* = 42 (*Osbp*, *Atg16*) cells were measured. (I–K) *Vap33*^*Δ448*^ *Osbp*^*Δ*^ double mutant enterocyte cells (K) possess similar levels of Sec61α-GFP (green) compared to *Vap33*^*Δ448*^ (I) single mutant enterocytes and increased levels of Sec61α-GFP compared to *Osbp*^*Δ*^ (J) single mutant cells. (L) Quantification of Sec61α-GFP intensity in enterocytes of each mutant genotype normalized to neighboring control cells. *n* = 29 (Ctrl), *n* = 13 (*Vap33*), *n* = 7 (*Osbp*), and *n* = 9 (*Vap33*, *Osbp*) cells were measured. (M–O) *Start1*^*Δ*^ *Atg9*^*D51*^ double mutant enterocyte cells (O) possess increased levels of Sec61α-GFP (green) compared to either *Start1*^*Δ*^ (M) or *Atg9*^*D51*^ (N) single mutant cells. *Start1*^*Δ*^ single mutant cells possess similar Sec61α-GFP levels as neighboring control cells. (P) Quantification of Sec61α-GFP intensity in enterocytes of each mutant genotype normalized to neighboring control cells. *n* = 102 (Ctrl), *n* = 25 (*Start1*), *n* = 39 (*Atg9*), and *n* = 38 (*Start1*, *Atg9*) cells were measured. (Q–S) *Osbp*^*Δ*^ *Start1*^*Δ*^ double mutant enterocyte cells (S) possess increased levels of Sec61α-GFP (green) compared to either *Osbp*^*Δ*^ (Q) or *Start1*^*Δ*^ (R) single mutant cells. . (T) Quantification of Sec61α-GFP intensity in enterocytes of each mutant genotype normalized to neighboring control cells. *n* = 19 (Ctrl), *n* = 5 (*Osbp*), *n* = 9 (*Start1*), and *n* = 5 (*Osbp*, *Start1*) cells were measured. Animals were staged 2 h APF. (E-G), (I-K), (M-O) and (Q-S) mutant cells lacking RFP (red) were indicated by white dotted lines. Scale bars in (B), (E-G) and insets, (I-K) and insets, (M-O) and insets and (Q-S) and insets represent 20 μm, and scale bar in (B) inset represents 5 μm. Insets in (B) are from indicated rectangles (white rectangle = mutant cell, yellow rectangle = control cell). White arrows in (B) indicate co-localization of Cathepsin L, Osbp and Hrs. Data are presented as mean ± SEM. n.s. = not significant, **p < 0.01, ***p < 0.001, and ****p < 0.0001 from one-way ANOVA corrected by Tukey’s post hoc test and unpaired, two-tailed *t* test. Each data point represents one mutant cell/neighboring cell. Representative of 3 or more independent biological experiments from ≥3 different animals.

The lipid transfer oxysterol-binding protein (OSBP) interacts with Vap-A/B to facilitate ER association with lysosomes.^20,21^ This prompted us to investigate the role of OSBP in ER to lysosome trafficking. We tagged the N terminus of *Drosophila* Osbp with 3×FLAG-V5 by CRISPR-Cas9 gene editing (Figure 3A). Interestingly, larger *Atg9* mutant enterocyte cells exhibit increased Osbp intensity and ESCRT-0 Hrs co-localization with Cathepsin L puncta compared to the smaller neighboring control cells 2 h APF (Figures 3B and 3C). In addition, Osbp and Hrs co-localize on the periphery of lysosomal Cathepsin L puncta (Figures 3B’ and 3D). By contrast, *Vap33* mutant enterocytes exhibit similar Osbp co-localization and intensity with lysosomal Cathepsin L puncta compared to neighboring control cells 2 h APF (Figures S2N and S2O). These data suggest that loss of macroautophagy promotes Osbp and ESCRT Hrs localization with lysosomes, while *Vap33* has little influence on Osbp association with the lysosome.

The localization of Osbp with lysosomes in the absence of macroautophagy prompted us to investigate whether Osbp is required for *Atg* gene-independent ER clearance. Therefore, we created a 1.8 kb deletion *Osbp*^*Δ*^ mutant strain by CRISPR-Cas9 gene editing that removes most of the Osbp open reading frame (Figures S3A and S3B). Surprisingly, *Osbp*^*Δ*^ single mutant enterocytes exhibit similar Sec61α-GFP ER marker intensity compared to neighboring control cells (Figures 3E and 3H). Importantly, *Osbp*^*Δ*^ and *Atg16*^*Δ*^ double mutant enterocytes exhibit increased Sec61α-GFP levels compared to either *Osbp*^*Δ*^ or *Atg16*^*Δ*^ single mutant cells (Figures 3E-3H). Furthermore, *Osbp* and *Atg16* double mutant enterocytes exhibit decreased ss-mKate2-KDEL ER sensor puncta co-localization with LysoTracker, and increased large ss-mKate2-KDEL co-localization with ss-pHluorin-KDEL puncta, compared to *Atg16* single mutant enterocytes (Figures S3C-S3E). Consistent with these results, Osbp has minor influence on ATP5A, Ref(2)p and Atg8a puncta (Figures S3F-S3J).

We next investigated whether Vap33 and Osbp function in the same pathway to regulate ER clearance. *Vap33*^*Δ448*^ and *Osbp*^*Δ*^ double mutant RFP-negative enterocytes possess similar ER-localized Sec61α-GFP intensity compared to *Vap33* single mutant cells, and increased Sec61α-GFP intensity compared to *Osbp* single mutant enterocytes 2 h APF (Figures 3I-3L). These data indicate that Vap33 and Osbp function in the same pathway to influence ER clearance.

Like OSBP, the lysosomal transmembrane StAR-related lipid transfer protein 3 (STARD3) interacts with Vap proteins to regulate cholesterol transport from the ER to lysosomes.^22^ Since *Drosophila* steroidogenic acute regulatory protein-like encoding gene Start1 is most similar to STARD3, we next investigated the function of *Start1* in ER clearance. We generated a *Start1*^*Δ*^ mutant strain by CRISPR-Cas9 gene editing (Figure S4A). Similar to *Osbp*, *Start1*^*Δ*^ single mutant enterocytes exhibit similar Sec61α-GFP intensity compared to neighboring control cells (Figures 3M and 3P). Importantly, *Atg9* and *Start1* double mutant cells possess increased Sec61α-GFP intensity compared to either *Start1* or *Atg9* single mutant cells (Figures 3M-3P). Since neither *Osbp* nor *Start1* has a robust influence on Sec61α-GFP levels, we tested whether *Osbp* and *Start1* function in a redundant manner to regulate *Atg*-independent ER clearance. Interestingly, *Osbp* and *Start1* double-mutant enterocytes possess increased Sec61α-GFP intensity compared to either *Osbp* or *Start1* single-mutant cells (Figures 3Q-3T). Similar to *Osbp* and *Atg16* double mutant enterocyte cells, *Atg9* and *Start1* double mutant enterocytes possess decreased ss-mKate2-KDEL ER sensor puncta co-localization with LysoTracker, and increased co-localization of ss-mKate2-KDEL and ss-pHluorin-KDEL puncta, compared to *Atg9* single mutant enterocyte cells (Figures S4B-S4D). Combined, these data indicate that Vap33, Osbp, and Start1 function in a pathway to regulate macroautophagy-independent ER clearance.

**Figure 4.**
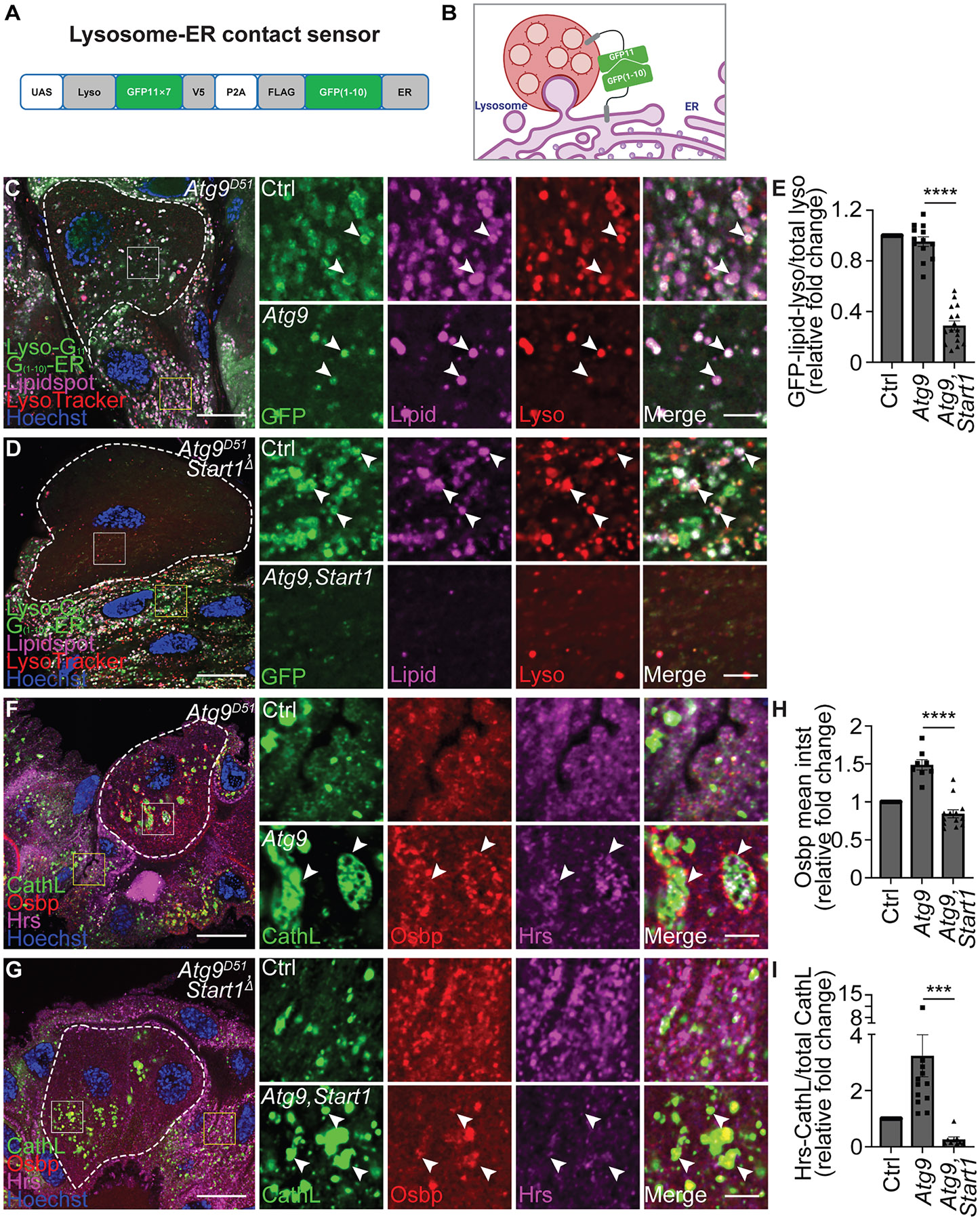
Start1 regulates lysosome and ER contact and Osbp association with the lysosome **(A)** Schematic of ER and lysosome contact sensor. (B) Schematic of lysosome and ER proximity detection using the Lyso-GFP_11_×7-V5-P2A-FLAG-GFP(_1–10_)-ER sensor. (C and D) *Atg9*^*D51*^ *Start1*^*Δ*^ double mutant enterocytes (D, white dotted line, larger cell compared to neighboring control cells) that express Lyso-GFP_11_×7-V5-P2A-FLAG-GFP(_1-10_)-ER (Lyso-G_11_-G(_1-10_)-ER) in all cells exhibit decreased GFP co-localization with Lipidspot (magenta) and LysoTracker (red) puncta compared to *Atg9*^*D51*^ single mutant enterocytes (C, white dotted line, larger cell compared to neighboring control cells). (E) Quantification of the ratio of co-localized GFP, Lipidspot, and LysoTracker puncta of total LysoTracker puncta in enterocytes of each mutant genotype normalized to neighboring control cells. *n* = 29 (Ctrl), *n* = 13 (*Atg9*), and *n* = 16 (*Atg9*, *Start1*) cells were measured. (F–G) *Atg9*^*D51*^ *Start1*^*Δ*^ double mutant enterocyte cells (G, white dotted line, larger cell compared to neighboring control cells) exhibit decreased V5-3×FLAG-Osbp (Osbp, red) intensity and decreased Osbp and Hrs (magenta) puncta surrounding Cathepsin L puncta (CathL, green) compared to *Atg9*^*D51*^ single mutant enterocyte cells (F, white dotted line, larger cell compared to neighboring control cells). (H) Quantification of Osbp mean intensity in enterocytes of each mutant genotype normalized to neighboring control cells. *n* = 21 (Ctrl), *n* = 8 (*Atg9*), and *n* = 13 (*Atg9*, *Start1*) cells were measured. (I) Quantification of the ratio of co-localized Hrs and Cathepsin L puncta of total Cathepsin L puncta in enterocytes of each mutant genotype normalized to neighboring control cells. *n* = 23 (Ctrl), *n* = 9 (*Atg9*), and *n* = 14 (*Atg9*, *Start1*) cells were measured. Animals were staged 2 h APF. Scale bars in (C), (D), (F) and (G) represent 20 μm, and scale bars in all insets represent 5 μm. All insets are from indicated rectangles (white rectangle = mutant cell, yellow rectangle = control cell). White arrows in (C-D) indicate co-localized GFP, Lipidspot and LysoTracker puncta. White arrows in (F-G) indicate co-localized Cathepsin L, Osbp and Hrs. Data are presented as mean ± SEM. ***p < 0.001 and ****p < 0.0001 from one-way ANOVA corrected by Tukey’s post hoc test and unpaired, two-tailed *t* test. Each data point represents one mutant cell/neighboring cell. Representative of 3 or more independent biological experiments from ≥3 different animals.

The ER is dynamic, contacts multiple organelles, and plays important roles in organelle health and homeostasis.^18,19^ ER contact with late endosomes or lysosomes facilitates the exchange of lipids and other nutrients between these organelles.^23^ Since we observed ER delivery to lysosomes in the absence of *Atg* genes, we explored whether ER and lysosome contact influences ER transfer to the lysosome. We constructed a split-green fluorescent protein (GFP) sensor to quantify ER and lysosome contact based on a related inter-organelle sensor in human cells^24^ that was validated in *Drosophila*.^25^ Specifically, the C terminus of *Drosophila* lysosomal transmembrane protein TMEM192 was fused with seven repeats of GFP11 and V5, and the ER targeting motif N terminus was derived from the tail-anchored human ER protein Cytochrome *b*5^26^ that was fused with GFP_(1-10)_ and FLAG. The two GFP modules were separated by a ribosome skipping motif (P2A) and the construct was placed under control of a UAS promoter (UAS-Lyso-GFP11×7-V5-P2A-FLAG-GFP_(1-10)_-ER) (Figures 4A and 4B). To diminish potential artificial enhancement of contact because of overexpression, we used the temperature-controlled GAL80^ts^-GAL4 system, which has mild expression at restrictive temperature (19°C) for experimental analysis. Importantly, macroautophagy-deficient *Atg9* mutant enterocytes exhibit GFP-positive ER and lysosome membrane contact site reporter puncta and comparable co-localization with LysoTracker and neutral lipid puncta compared to neighboring control cells (Figures 4C and 4E). These results suggest that the ER contacts lysosomes that contain neutral lipids in cells lacking macroautophagy.

Since STARD3 regulates lysosome and ER contact,^22,27^ we tested if fly Start1 regulates lysosome and ER contact. Interestingly, lysosome and ER membrane contact site co-localization with neutral lipid-containing lysosomes of *Atg9* and *Start1* double mutant enterocytes is decreased compared to *Atg9* single mutant enterocytes (Figures 4C-4E). Importantly, *Atg9* and *Start1* double mutant enterocytes exhibit decreased Osbp intensity, and Hrs and Cathepsin L puncta co-localization compared to *Atg9* single mutant enterocytes, even though Cathepsin L is abundant in *Atg9* and *Start1* double mutant cells (Figures 4F-4I).

### Cholesterol transfer from the ER to the lysosome is associated with *Atg*-independent ER clearance

**Figure 5.**
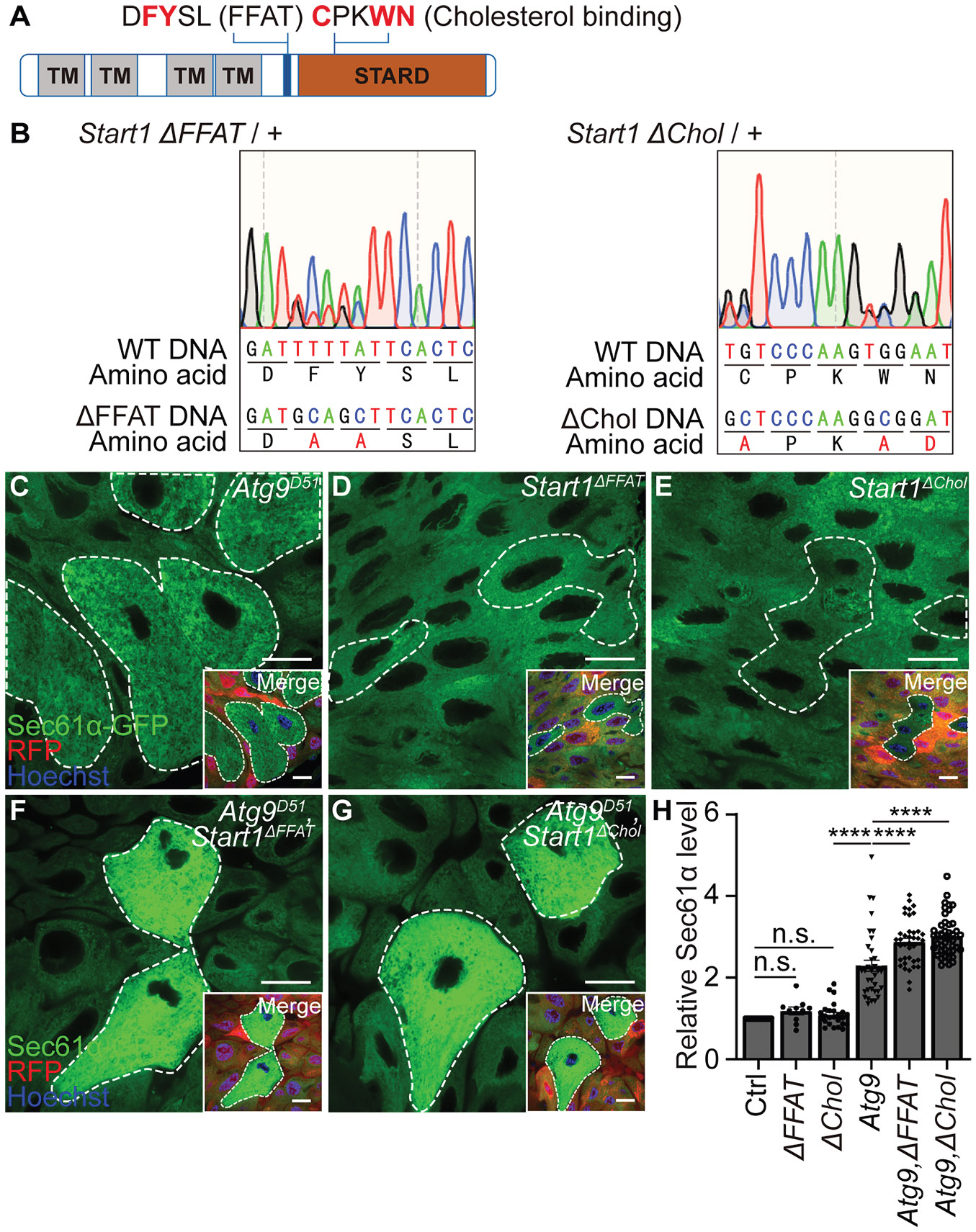
Start1 FFAT and cholesterol-binding domains are required for macroautophagy-independent ER clearance (A) Schematic diagram of Start1 domains. (B) Diagram of the heterozygous Vap33 interacting mutant *Start1* (*Start1*^*ΔFFAT*^/+) and cholesterol binding mutant *Start1* (*Start1*^*ΔChol*^/+) mutant allele. (C–G) Either *Atg9*^*D51*^ *Start1*^*ΔFFAT*^ (F) or *Atg9*^*D51*^ *Start1*^*ΔChol*^ (G) double mutant enterocyte cells possess enhanced Sec61α-GFP levels (green) compared to either *Atg9*^*D51*^ (C), *Start1*^*ΔFFAT*^ (D), or *Start1*^*ΔChol*^ (E) single mutant cells. *Start1*^*ΔFFAT*^ and *Start1*^*ΔChol*^ single mutant enterocytes possess similar Sec61α-GFP levels compared to each relative neighboring control cells. Sec61α-GFP was expressed in all cells and mutant cells lack of RFP (red). (H) Quantification of Sec61α-GFP intensity in each genotype normalized to neighboring control cells. *n* = 153 (Ctrl), *n* = 10 (*ΔFFAT*), *n* = 25 (*ΔChol*), *n* = 36 (*Atg9*), *n* = 38 (*Atg9*, *ΔFFAT*) and *n* = 44 (*Atg9*, *ΔChol*) cells were measured. Animals were staged 2 h APF. Scale bars in (C-G) and insets represent 20 μm. Data are presented as mean ± SEM. n.s. = not significant, ****p < 0.0001 from one-way ANOVA corrected by Tukey’s post hoc. Each data point represents one mutant cell/ neighboring cell. Representative of 3 or more independent biological experiments from ≥3 different animals.

To investigate the function of Start1 in ER and lysosome contact and cholesterol binding during macroautophagy-independent ER clearance, we generated two mutations in Start1 by gene editing (Figures 5A and 5B). The *Start1*^*ΔFFAT*^ mutant was used to interrogate ER and lysosome contact and ER clearance since the STARD3 FFAT domain is known to function as an ER and lysosome tether.^27^ In addition, the *Start1*^*ΔChol*^ mutant was used to study cholesterol binding and potential transfer from the ER to the lysosome.^22,28^ Either *Start1*^*ΔFFAT*^ or *Start1*^*ΔChol*^ single mutant enterocyte cells possess similar Sec61α-GFP intensity compared to relative neighboring control cells (Figures 5D and 5E and 5H). By contrast, either *Atg9*^*D51*^*Start1*^*ΔFFAT*^ or *Atg9*^*D51*^*Start1*^*ΔChol*^ double mutant cells possess increased Sec61α-GFP intensity compared to *Atg9*^*D51*^ single mutant cells (Figures 5C and 5F-5H). Importantly, either *Atg9*^*D51*^*Start1*^*Δ*^, *Atg9*^*D51*^*Start1*^*ΔFFAT*^, or *Atg9*^*D51*^*Start1*^*ΔChol*^ double mutant cells exhibit similar amounts of active Cathepsin B cleaved fluorescent Magic Red puncta compared to *Atg9*^*D51*^ single mutant cells (Figures S5A-S5E), indicating that lysosomes possess equivalent Cathepsin B function in these mutant cells. In addition, *Atg9*^*D51*^*Start1*^*ΔChol*^ double mutant cells exhibit slightly increased ER and lysosome contact compared to either *Atg9*^*D51*^*Start1*^*Δ*^ or *Atg9*^*D51*^*Start1*^*ΔFFAT*^ double mutant cells (Figures S5F-S5J). These data suggest that Start1 influences lysosomal Osbp localization and that Vap33 interacts with the Start1 FFAT motif to facilitate ER and lysosome proximity and ER clearance. Interestingly, Start1 cholesterol binding is also required for ER clearance that is independent of macroautophagy *Atg* gene function.

**Figure 6.**
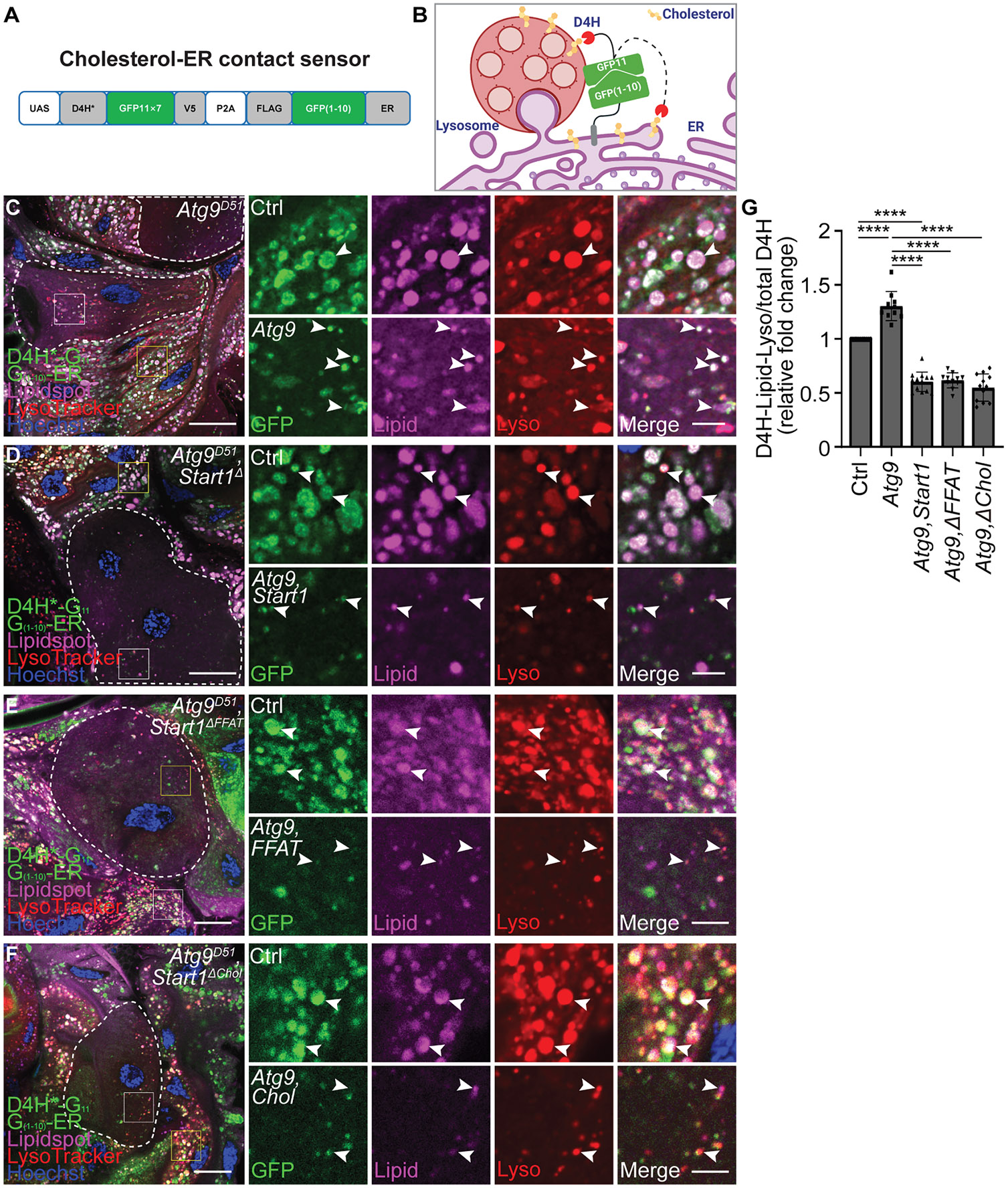
Start1 regulates Cholesterol-ER contact independent of macroautophagy (A) Schematic of Cholesterol-ER contact sensor. (B) Schematic of Cholesterol and ER proximity detection using the D4H*-GFP_11_×7-V5-P2A-FLAG-GFP(_1-10_)-ER sensor. (C–F) *Atg9*^*D51*^*Start1*^*Δ*^ (D), *Atg9*^*D51*^*Start1*^*ΔFFAT*^ (E) and *Atg9*^*D51*^*Start1*^*ΔChol*^ (F) double mutant enterocytes (white dotted line, larger cell compared to neighboring control cells) that express D4H*-GFP_11_×7-V5-P2A-FLAG-GFP_(1-10)_-ER (D4H*-G_11_-G_(1-10)_-ER) in all cells exhibit decreased GFP co-localization with Lipidspot (magenta) and LysoTracker (red) puncta compared to *Atg9*^*D51*^ (C) single mutant enterocytes (white dotted line, larger cell compared to relative neighboring control cells). (G) Quantification of the ratio of co-localized GFP, Lipidspot, and LysoTracker puncta of total LysoTracker puncta in enterocytes of each mutant genotype normalized to neighboring control cells. *n* = 50 (Ctrl) and *n* = 9 (*Atg9*), *n* = 18 (*Atg9 Start1*), *n* = 11 (*Atg9 ΔFFAT*) and *n* = 12 (*Atg9 ΔChol*) cells were measured. Animals were staged 2 h APF. Scale bars in (C-F) represent 20 μm, and scale bars in (C-F) insets represent 5 μm. All insets are from indicated rectangles (white rectangle = mutant cell, yellow rectangle = control cell). White arrows in (C-F) indicate co-localization of GFP, Lipidspot and LysoTracker puncta. Data are presented as mean ± SEM. n.s. = not significant, ****p < 0.0001 from one-way ANOVA corrected by Tukey’s post hoc. Each data point represents one mutant cell/ neighboring cell. Representative of 3 or more independent biological experiments from ≥3 different animals.

The lysosome is a lipid salvage center, and the exchange of lipids between organelles occurs by organelle contact.^19^ In addition, the interactions between Vap-A/B with either OSBP or STARD3 facilitate both lysosome and ER contact and cholesterol transport from ER to lysosomes.^20,22^ To investigate the relationships between cholesterol, ER, and lysosomes, we generated a split-GFP cholesterol and ER contact sensor. The C terminus of D4H*, which was derived from the cholesterol-binding domain of Perfringolysin O, was fused with GFP11×7-V5, and the N-terminal ER transmembrane targeting motif of Cytochrome *b*5 was fused with FLAG-GFP _(1-10)_. The two modules were separated by a P2A and placed under control of a UAS promoter (UAS-D4H*-GFP_11_×7-V5-P2A-FLAG-GFP_(1-10)_-ER) (Figures 6A and 6B). Since the ER forms contacts with neutral lipid-containing lysosomes in an *Atg* gene-independent manner, we tested if macroautophagy influences ER contact with organelles that contain cholesterol, and how Start1 influences lipid localization. *Atg9* mutant enterocyte cells exhibit smaller ER and cholesterol contact GFP puncta, but possess increased co-localization with Lipidspot and LysoTracker puncta compared to neighboring control cells (Figures 6C and 6G). Importantly, *Atg9*^*D51*^*Start1*^*Δ*^, *Atg9*^*D51*^*Start1*^*ΔFFAT*^, and *Atg9*^*D51*^*Start1*^*ΔChol*^ double mutant enterocytes exhibit decreased ER and cholesterol contact GFP puncta co-localization with Lipidspot and LysoTracker puncta compared to *Atg9* single mutant enterocytes (Figures 6C-6G). These data indicate that Start1 is required for ER contact with lysosomes that contain cholesterol, and that this association is independent of macroautophagy.

Niemann-Pick C1 (NPC1) regulates lysosomal cholesterol export.^20^ Therefore, we tested if *Drosophila* Npc1a impacts cholesterol levels and ER clearance. Consistent with previous work, *Npc1a* loss-of-function mutant enterocytes possess increased Filipin puncta density, a marker of cholesterol, and Filipin puncta partially co-localize with Cathepsin L puncta compared to neighboring control cells (Figures S6A and S6B). However, *Npc1a* mutant enterocytes possess similar Osbp intensity compared to neighboring control cells (Figures S6C and S6D). In addition, *Npc1a* mutant enterocytes possess similar Sec61α-GFP intensity compared to neighboring control cells (Figures S6E and S6F). These data indicate that Osbp lysosomal localization is independent of *Npc1a*, and that *Npc1a* is not required for ER clearance.

**Figure 7.**
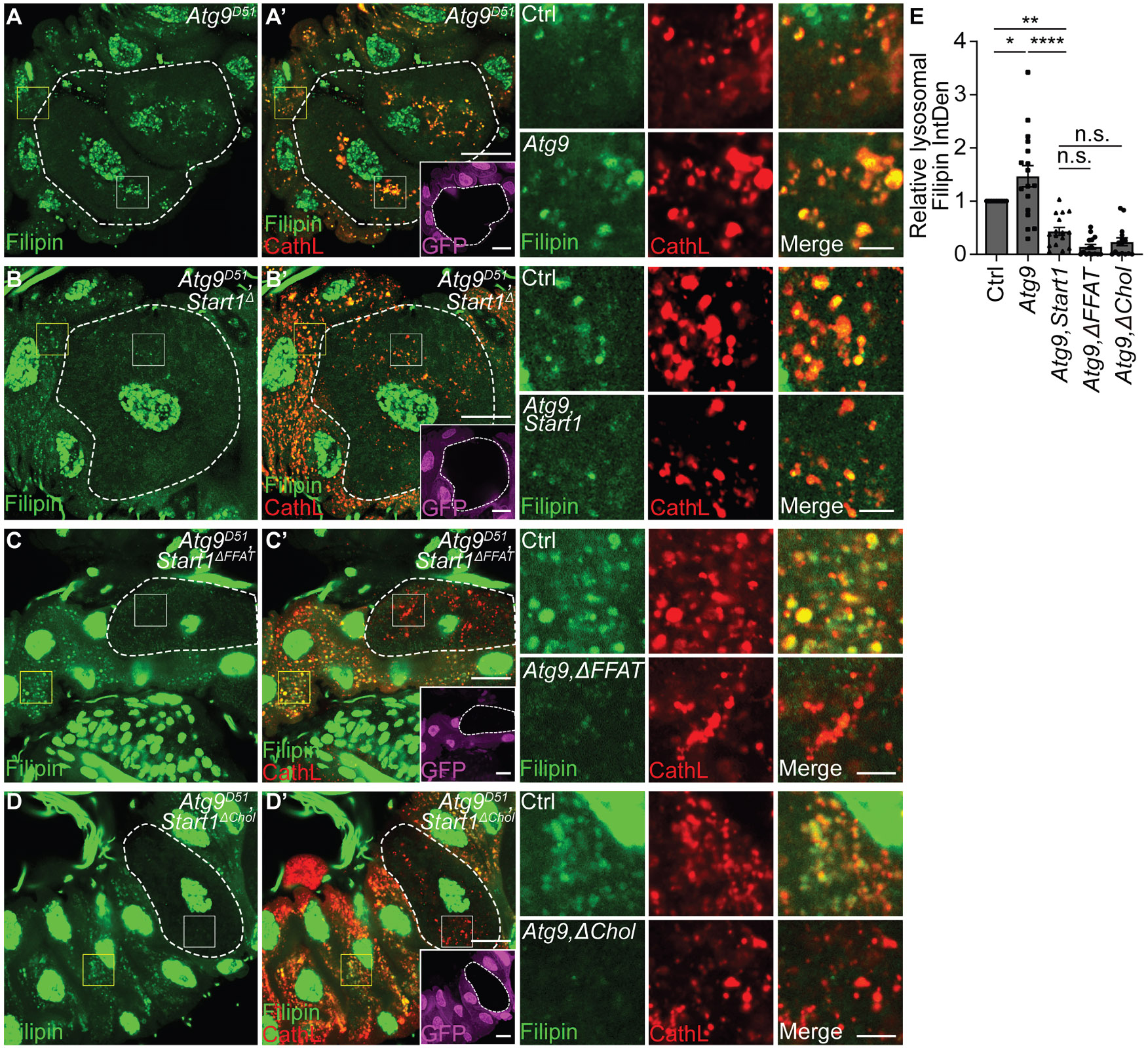
Start1 affects lysosomal cholesterol content (A–D) *Atg9*^*D51*^*Start1*^*Δ*^ (B), *Atg9*^*D51*^*Start1*^*ΔFFAT*^ (C), and *Atg9*^*D51*^*Start1*^*ΔChol*^ (D) double mutant enterocytes (white dotted line, lacking GFP (magenta)) exhibit decreased Filipin (green) density and co-localization with Cathepsin L (CathL, magenta) compared to *Atg9*^*D51*^ single mutant cells (A). (E) Quantification of average Filipin integrated density (IntDen) co-localization with Cathepsin L puncta in enterocytes of each mutant genotype normalized to neighboring control cells. *n* = 63 (Ctrl), *n* = 17 (*Atg9*), *n* = 16 (*Atg9 Start1*), *n* = 15 (*Atg9 ΔFFAT*), and *n* = 15 (*Atg9 ΔChol*) cells were measured. Animals were staged 2 h APF. Scale bars in (A-D) represent 20 μm, and scale bars in insets represent 5 μm. All insets are from indicated rectangles (white rectangle = mutant cell, yellow rectangle = control cell). Data are presented as mean ± SEM. n.s. = not significant, *p < 0.05, **p < 0.01, and ****p < 0.0001 from one-way ANOVA corrected by Tukey’s post hoc. Each data point represents one mutant cell/neighboring cell. Representative of 3 or more independent biological experiments from ≥3 different animals.

Our data indicate that ER and lysosomal contact proteins influence *Atg*-independent ER clearance and lysosomal cholesterol content. To further test this model, we examined whether macroautophagy affects lysosomal cholesterol content and how Start1 influences lysosomal cholesterol. *Atg9* mutant enterocytes exhibit elevated Filipin puncta density co-localization with Cathepsin L compared to neighboring control cells (Figures 7A and 7E). By contrast, either *Atg9*^*D51*^*Start1*^*Δ*^*, Atg9*^*D51*^*Start1*^*ΔFFAT*^, *or Atg9*^*D51*^*Start1*^*ΔChol*^ double mutant enterocytes possess decreased Filipin puncta density and co-localization with lysosomal Cathepsin L compared to *Atg9* single mutant enterocyte cells (Figures 7B-7E). Combined, these data indicate that Start1 regulates cholesterol transport from the ER to lysosomes by inter-organelle contact.

## DISCUSSION

This study reveals how a single cell utilizes two parallel pathways to clear the ER during development. Consistent with previous work,^5,6,11^ ESCRT machinery is required for *Atg* gene-independent ER removal. Since previous studies of *Atg* gene-independent ER clearance were conducted in yeast and cultured cells under chronic ER stress,^9,10^ this study provides evidence that this process occurs under physiological conditions in an animal during development.

The relationship between the ER and lysosomes is important for cholesterol homeostasis and health.^29-31^ We discovered that cholesterol transport from ER to lysosomes is associated with the selection of ER for clearance that is independent of macroautophagy *Atg* genes. Significantly, we identify Vap33 and Osbp as regulators of ER and lysosome contact for ER delivery to lysosomes for clearance, and this is consistent with previous studies of lysosome damage and repair in cell lines, indicating that Vap and Osbp family members serve as tethers that promote ER and lysosome proximity.^32^ In contrast to previous work, we show that Vap33 and Osbp also function with Start1 to transfer cholesterol and ER to lysosomes for degradation, and that this occurs in an *Atg* gene-independent manner during animal development. It is possible that ER cholesterol levels influence ER and lysosome contact, as it has been previously reported that cholesterol depletion promotes ER contact with mitochondria.^33^ In addition, cholesterol may influence the morphology of the ER to promote delivery to lysosomes.

Multiple forms of autophagy exist, yet little is known about why different mechanisms exist to deliver substrates to lysosomes. Chaperone-mediated autophagy is used to target proteins to lysosomes based on a specific peptide sequence,^34^ thus making this process different from the other forms of autophagy. By contrast, both macroautophagy-dependent and -independent mechanisms exist to clear the ER. Our data could indicate that *Atg* gene-independent ER delivery to lysosomes provides a backup mechanism to macroautophagy, but it is also possible that this ER has properties that require an alternative mechanism for transfer to lysosomes. Since macroautophagy-dependent^8^ and -independent ER clearance pathways exist in the same physiological context during development, future studies of this model will advance our understanding of how different ER clearance pathways are coordinated and function to maintain cell health.

### Limitations of the study

Further investigations will be needed to understand if Vap33, Osbp, and Start1 regulate macroautophagy-independent ER clearance in other cell contexts, including human cells. It is also important to rigorously investigate the influence of ER and lysosome contact on cholesterol dynamics in other cell and tissue contexts. Studies of ER and other organelle dynamics under physiological conditions in a complex animal tissue with multiple cell types present experimental challenges, such as biochemical analysis of purified cell and organelle populations. Thus, it is important to resolve mechanistic issues raised by this study, including but not limited to how cholesterol influences ER and lysosome contact to influence macroautophagy-independent clearance. Finally, it is critical to better resolve the relationship between macroautophagy-dependent and -independent ER clearance, and better understand how cholesterol, ER subdomains, and likely other factors influence ER shape and clearance by different mechanisms.

## STAR★METHODS

### EXPERIMENTAL MODEL AND STUDY PARTICIPANT DETAILS

*Drosophila melanogaster* strains and genotypes used in this study are listed in the key resources table and Table S2. The animals used in this study were of both genders unless noted for specific mutant genotypes. Animals were analyzed as either feeding third-instar larvae or 2 h after puparium formation as noted. We did not observe any influence of gender on results. Flies were reared at 25°C on standard cornmeal/molasses/agar media.

### METHOD DETAILS

#### *Drosophila melanogaster* stocks

*Atg9*^*D51*^ was a gift from G. Chen.^35^ *Vps25*^*N55*^ was a gift from Andreas Bergmann. *Atg16*^*Δ*^, *Atg8a*^*Δ*^ and UAS-ss-pHluorin-mKate2-KDEL-V5 were generated in our previous studies.^8,14^

The *Osbp*^*Δ*^ loss-of-function strains were created by crossing vasa-Cas9; FRT82B with *Osbp* gRNA transgenic flies. The *Start1*^*Δ*^ loss-of-function strain was generated by crossing FRT42D; vasa-Cas9 with *Start1* gRNA. gRNA and screening primer sequences are provided in Table S1. Single F1 progeny of parental vasa-Cas9 and gRNA crosses were mated with virgin female double balancer chromosome flies, and F2 progeny were selected with G418 (25 mg/mL) against non-FRT (neomycin resistant) flies. Gene deletion mutants were identified by PCR and validated by DNA sequencing. Mutants were then backcrossed 5 times to remove Cas9/gRNA transgenes, and recombined with appropriate FRTs on the chromosome arm for analysis of each gene mutant.

The lysosome and ER contact sensor UAS-Lyso-GFP_11_×7-V5-P2A-FLAG-GFP_(1-10)_-ER was produced by combing full length *Drosophila* TMEM192 (NM_142386.4) for lysosomal targeting and a validated ER targeting C-terminal transmembrane domain peptide from rat Cytochrome *b*5 (CB5).^36,37^ Modified D4H*^20^ and CB5 were fused with split-GFPs to generate the UAS-D4H*-GFP_11_×7-V5-P2A-FLAG-GFP_(1-10)_-ER sensor. The entire coding region of both of these sensors flanked by 15 bp homologous arms (Table S1) were synthesized by IDT (San Diego, California) and assembled into a pUAST-attB vector (*Drosophila* Genomics Resource Center, 1419) using the In-Fusion HD Cloning Kit. Plasmid DNA was injected by Bestgene (Chino Hills, California) into a strain carrying attP2 landing sites, and stably integrated into the third chromosome of the *Drosophila* genome using phiC31 integrase.

#### Gene editing to tag Vap33 and osbp

*Vap33* and *Osbp* encoded proteins were tagged on their N-termini with V5-3×FLAG separated by a 15 amino acids polypeptide linker (GGGGSGLRSSRGPFE) by CRISPR-Cas9 gene editing. Start1 amino acids 226-DFYSL-236 were mutated to 226-DAASL-236 to generate *Start1*^*ΔFFAT*^ and 315-CPKWN-319 were mutated to 315-APKAD-319 to generate *Start1*^*ΔChol*^ by CRISPR-Cas9 gene editing. gRNAs were designed by using https://flycrispr.org/ and https://www.flyrnai.org/crispr3/web/ with low off-target scores and high frameshift scores, and were selected against off-targets. We used two gRNAs flanking either the *Vap33*, *Osbp* start codons, or *Start1* designated nucleic acid sites for gene editing. gRNA oligonucleotides were synthesized by IDT and subcloned into pCFD3.1-w-dU6:3 gRNA (Addgene, 123366). Guide RNA sequences are provided in Table S3. We designed 500–600 bp homologous arms for each gene, and all PAM sequences were silent mutations unless the PAM was located in the intron or 5′-UTR regions. Homologous directed repair donor templates were synthesized by IDT and subcloned into TOPO vectors (Invitrogen). gBlock sequences are provided in Table S1 gRNAs and donors were co-injected into vasa-Cas9 transgenic flies. Germline injection was conducted by Bestgene (Chino Hills, California). Progeny were collected and screened for precise insertions by DNA sequencing.

#### Induction of mosaic mutant cells

Mosaic mutant cell clones were induced as previously described.^38^ To induce loss-of-function mutant cell clones, embryos were collected for 5 h and heat shocked at 37°C for 1 h.

#### Immunolabeling and microscopy

Intestines were dissected from staged animals in PBS, fixed with 4% paraformaldehyde (PFA) in PBS 0.3% Triton X-100 (PBST), and blocked with normal goat serum (NGS) for 2 h before incubating with primary antibodies in 0.3% PBST with 5% goat serum. We used rabbit anti-Cathepsin L (1:200), mouse anti-V5 (1:200), and guinea pig anti-Hrs (1:200) for immunostaining. All secondary antibodies were incubated for 2 h at room temperature. Hoechst 33342 dye was used to stain DNA. Intestines were mounted in VectaShield, and imaged using either a Zeiss LSM 700 or Nikon A1R HD25 confocal microscope. Zeiss LSM 700 images were obtained using a Plan-Apochromat 63x/1.40 Oil DIC M27 objective and Zeiss Zen Software. Zeiss LSM 980 Airyscan 2 images were obtained using a Plan-Apochromat 63x/1.40 Oil DIC M27 objective and Zeiss Zen Software. Nikon A1R HD25 images were obtained using a CFI Plan Apochromat 60x/1.4 Oil DIC objective and NIS-Elements Viewer software. Images collected with the Nikon A1R HD25 were further magnified by 2.88. Images were deconvoluted using NIS-Elements C software and processed with Fiji.^39^

#### Contact sensor expression

Embryos derived from crosses expressing either the NP1-GAL4 (Myo31DFNP0001), Tub-GAL80^ts^, UAS-Lyso-GFP_11_×7-V5-P2A-FLAG-GFP_(1-10)_-ER sensor or the D4H*-GFP_11_×7-V5-P2A-FLAG-GFP_(1-10)_-ER sensor were laid for 0–5 h at 25°C, heat shocked at 37°C for 1 h to induce mosaic cell clones, and cultured at the restrictive 19°C temperature until exhibiting similar cuticle color and buoyancy to pupae cultured at 25°C 2 h APF.

#### LysoTracker and lipidspot staining

Fly intestines expressing either ss-pHluorin-mKate2-KDEL-V5, Lyso-GFP_11_×7-V5-P2A-FLAG-GFP_(1-10)_-ER or D4H*-GFP_11_×7-V5-P2A-FLAG-GFP_(1-10)_-ER were dissected either 2 h APF if cultured at 25°C or until exhibiting similar cuticle color and buoyancy compared to 25°C pupae at 2 h APF if cultured at 19°C in 1×PBS. Intestines were incubated in either LysoTracker Deep Red (1:700) and DNA stain Hoechst (1:25) in 1×PBS (pH 7.4) or with LysoTracker red DND-99 (1:1000), Lipidspot 610 (1:600) and Hoechst (1:25) in 1×PBS (pH 7.4) at room temperature for 10–15 min. The intestines were mounted in VectaShield and immediately imaged by confocal microscopy.

#### Magic Red staining

Fly intestines were dissected 2 h after puparium formation in 1×PBS. Intestines were incubated in Magic Red (1:125, Cathepsin B Assay kit) and DNA stain Hoechst (1:25) in 1×PBS (pH 7.4) at room temperature for 10–15 min. The intestines were mounted in VectaShield and immediately imaged by confocal microscopy.

#### Filipin staining

Fly intestines were dissected 2 h after puparium formation in 1×PBS and fixed with 4% PFA in 1×PBS for 1 h at room temperature with rotation. Intestines were transferred to 5% NGS in 0.3% PBST, and incubated with Cathepsin L antibody in 5% NGS in 0.3% PBST at 4°C overnight. Then intestines were washed with 1×PBS 5 times to diminish Triton X-100’s influence on Filipin staining. Filipin III was diluted (1:600) with secondary antibody (1:200) in 1×PBS. Intestines were incubated with Filipin solution for 2 h at room temperature in the dark.

#### Transmission electron microscopy

Intestines were dissected in PBS 2 h APF, fixed over night at 4°C in 2.5% glutaraldehyde and 2% paraformaldehyde in 0.1 M sodium cacodylate buffer, pH 7.4, osmicated, and washed in distilled water as previously described.^38^ Preparations were stained *en bloc* in 1% uranyl acetate and dehydrated through a graded series of ethanol series, treated with propylene oxide and embedded in SIP-pon/Araldite. Ultrathin sections of the anterior region of the midgut of the intestine were collected and stained with uranyl acetate and lead citrate. For each genotype, at least 3 intestines were embedded and sectioned for image acquisition and quantification. Imaging was performed using a Phillips CM10 TEM.

### QUANTIFICATION AND STATISTICAL ANALYSIS

Fiji^39^ was used to quantify immunofluorescence intensity and puncta in images of intestine cells. *p*-values were calculated using either a two-tailed unpaired *t* test, a Fisher’s LSD test, or a One-Way ANOVA corrected by Tukey’s post-hoc test by Graphpad Prism 5 (https://www.graphpad.com/scientific-software/prism/). The number (n) of samples analyzed by immunostaining represents number of enterocytes cells from at least 4 independent animals from each genotype. No animals were excluded from statistical analyses, experiments were not randomized, and the investigators were not blinded. All error bars are SEM.

## Supplementary Material

1

2

3

Supplemental information can be found online at https://doi.org/10.1016/j.celrep.2026.117537.

## Figures and Tables

**Table:** KEY RESOURCES TABLE

REAGENTS or RESOURCE	SOURCE	IDENTIFIER
Antibodies		
Cathepsin L	Abcam	ab58991
Hrs	Hugo Bellen	N/A
V5	Invitrogen	R960-25
Atg8a	Cell Signaling	13733
ATP5A	Abcam	ab14748
Ref(2)p	Abcam	ab178440
Anti-rabbit Alexa Fluor 488	Invitrogen	A-27034
Anti-mouse Alexa Fluor 546	Invitrogen	A-11030
Anti-guinea pig Alexa Fluor 647	Invitrogen	A-21450
Chemicals, peptides and recombinant proteins		
Normal goat serum	Life technologies	PCN5000
PBS	GIBCO	70011
Hoechst	Invitrogen	33342
Vectashield	Vector Laboratories	H-1200
Filipin III	Sigma-Aldrich	SAE0087
LysoTracker Deep Red	Invitrogen	L12492
LysoTracker red DND-99	Invitrogen	L7528
Lipidspot 610	Biotium	70069
Magic Red	Abcam	ab270772
In-Fusion HD Cloning Kit	Takara	639650
Experimental models: organisms/strains		
UAS-Lyso-GFP_11_×7-V5-P2A-FLAG-GFP(1-10)-ER (Attp2)	This study	N/A
UAS-D4H*-GFP_11_×7-V5-P2A-FLAG-GFP_(1-10)_-ER (Attp2)	This study	N/A
V5-3×FLAG-Vap33	This study	N/A
V5-3×FLAG-Osbp	This study	N/A
*Start1 ΔFFAT*	This study	N/A
*Start1 ΔChol*	This study	N/A
UAS-ss-pHluorin-mKate2-KDEL-V5 (Attp40, Attp2)	Eric Baehrecke	N/A
*Atg9* ^*D51*^	Guangchao Chen	N/A
*Atg16* ^*Δ*^	Eric Baehrecke	N/A
*Atg8* ^*Δ*^	This study	N/A
*Vps25* ^*N55*^	Andreas Bergmann	N/A
*Vps25* ^*A3*^	Bloomington *Drosophila* Stock Center	39633
*Lsn* ^*SS6*^	Bloomington *Drosophila* Stock Center	39631
*Vap33* ^*Δ448*^	Bloomington *Drosophila* Stock Center	39693
*Npc1A* ^*SK3*^	Fly stocks of National Institute of Genetics	M2L-2877
*Npc1A* ^*SK4*^	Fly stocks of National Institute of Genetics	M2L-2878
*Vps25* RNAi	Bloomington *Drosophila* Stock Center	26286
*Osbp gRNA*	Bloomington *Drosophila* Stock Center	92577
*Start1 gRNA*	Bloomington *Drosophila* Stock Center	82816
*Vap33* gRNA	Bloomington *Drosophila* Stock Center	81751
vasa-Cas9	Bloomington *Drosophila* Stock Center	56552
vasa-Cas9	Bloomington *Drosophila* Stock Center	51324
UAS::Cas9	Bloomington *Drosophila* Stock Center	58986
Tub-GAL80^ts^	Bloomington *Drosophila* Stock Center	7108
HsFlp, Ubi-nlsRFP, FRT19A	Bloomington *Drosophila* stock center	31416
hsFlp (D5)	Bloomington *Drosophila* stock center	55814
hsFlp; FRT42D, Ubi-nlsRFP	Eric Baehrecke	N/A
hsFlp; Ubi-nlsRFP, FRT40A	Eric Baehrecke	N/A
hsFlp; Ubi-nlsGFP, FRT40A	Eric Baehrecke	N/A
hsFlp;FRT82B, Ubi-nlsRFP	Eric Baehrecke	N/A
hsFlp; FRT42D	Eric Baehrecke	N/A
hsFlp;FRT82B	Eric Baehrecke	N/A
FRT19A; hsFlp (D5)	This study	N/A
hsFlp; FRT42D, Ubi-nlsRFP; FRT82B, Ubi-nlsRFP	This study	N/A
hsFlp, Ubi-nlsRFP, FRT19A;;FRT82B,Ubi-nlsRFP	This study	N/A
NP1-Gal4	Eric Baehrecke	N/A
Sec61α-GFP	Vienna *Drosophila* RNAi Center (VDRC)	318343
Recombinant DNA		
pCFD3.1-w-dU6:3gRNA	Addgene	123366
pUASTattB	Drosophila Genomics Resources Center	1419
pCR^™^ 2.1-TOPO^™^ TA vector	Invitrogen	450641
Oligonucleotides (See Table S1 for a list of oligonucleotides)		
Software and algorithms		
ImageJ	NIH	https://imagej.nih.gov/ij/
Prism	Graphpad Software	https://www.graphpad.com/scientific-software/prism/
ZEN	Zeiss	https://www.zeiss.com/microscopy/us/products/microscopesoftware/zen.html
NIS-Elements	Nikon	https://www.microscope.healthcare.nikon.com/products/software/nis-elements
Deposited data		
Mendeley	Eric Baehrecke	https://doi.org/10.17632/kksxbvrj6p.1

## Highlights

- ER clearance occurs in an *Atg* gene-independent manner during development
- Vap33, Osbp, and Start1 are required for ER clearance by lysosomes
- ER contact with lysosomes is important for *Atg*-independent ER clearance
- ER clearance promotes cholesterol transfer from the ER to lysosomes
