# Supplementary material for: Cholesterol transfer proteins promote Atg-independent ER clearance by lysosomes
Source: Cell Rep. Author manuscript; Available in PMC 2026 Jul 13. (PMC13358760; doi:10.1016/j.celrep.2026.117537)
Supplement: 1 [file NIHMS2190755-supplement-1.pdf]

Cell Reports, Volume *45*

Supplemental information

Cholesterol transfer proteins promote

Atg-independent ER clearance by lysosomes

Ruoxi Wang, Tina M. Fortier, Xiaofeng Sun, Fei Chai, Panagiotis D. Velentzas, and Eric H. Baehrecke

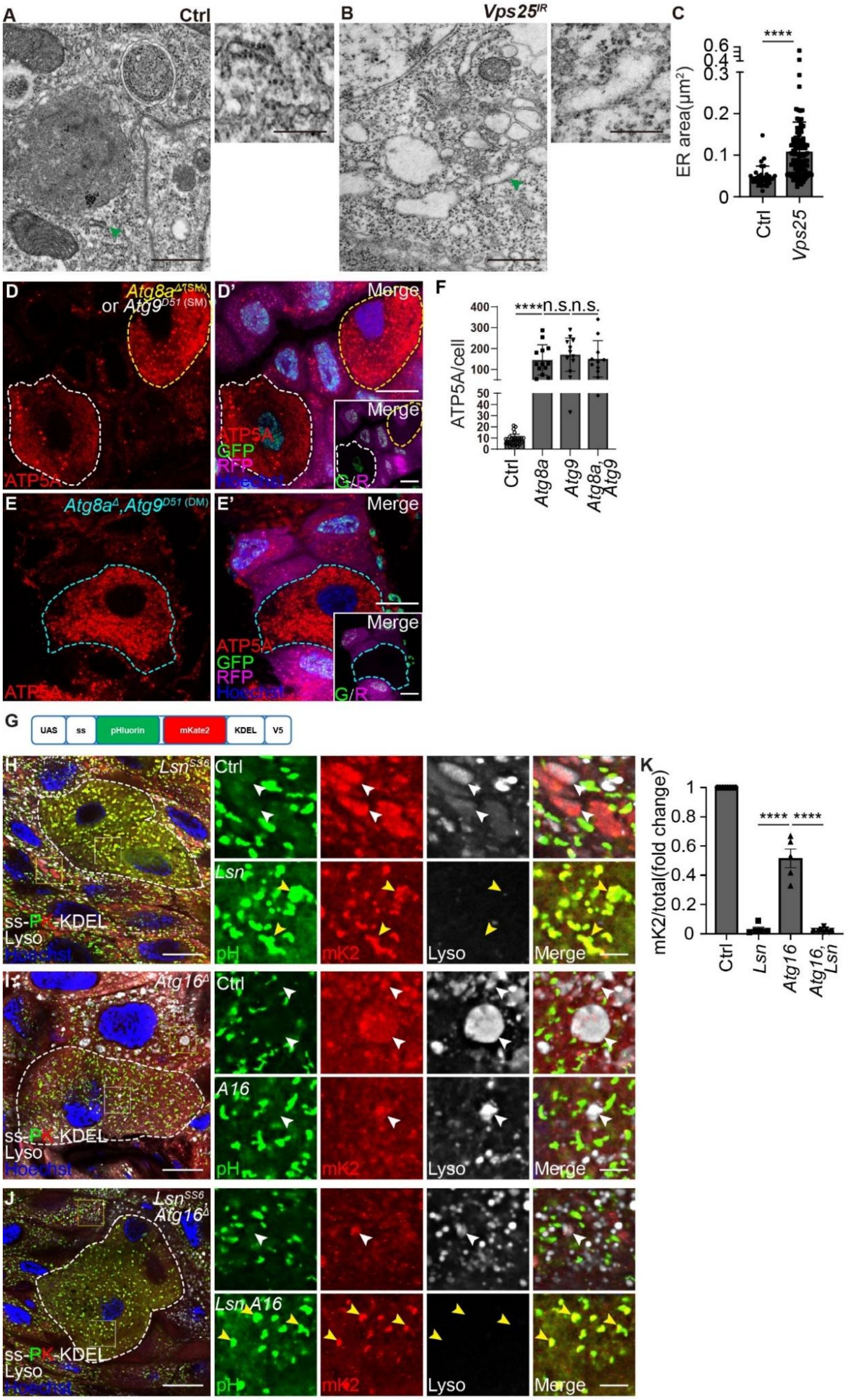

A
Ctrl
B
Vps25^IR
C
ER area(μm²)
****
Ctrl
Vps25
D
Atg8a^Δ (SM)
or Atg9^D51 (SM)
ATP5A
D'
Merge
ATP5A
GFP
RFP
Hoechst
G/R
F
ATP5A/cell
****
n.s.
n.s.
Ctrl
Atg8a
Atg9
Atg8a, Atg9
E
Atg8a^Δ,Atg9^D51 (DM)
ATP5A
E'
Merge
G
UAS
ss
pHluorin
mKate2
KDEL
V5
H
Lsn^SS6
ss-PK-KDEL
Lyso
Hoechst
Ctrl
Lsn
pH
mK2
Lyso
Merge
K
mK2/total(fold change)
****
****
Ctrl
Lsn
Atg16
Atg16, Lsn
I
Atg16^Δ
A16
J
Lsn^SS6
Atg16^Δ
Lsn A16

Figure S1. ESCRT regulates macroautophagy-independent ER clearance by lysosomes. Related to Figure 1.

(A-B) TEM images of *Luciferase* RNAi (Ctrl) and *Vps25* RNAi ($Vps25^{IR}$) that were expressed in all enterocytes driven by NP1-GAL4. $Vps25^{IR}$ cells exhibit dilated rough ER structures compared to control cells.

(C) Quantification of ER area. n = 31 (Ctrl), n = 100 (*Vps25*) ER structures were measured.

(D-E) $Atg8^{\Delta}$ $Atg9^{D51}$ double mutant enterocytes (labeled by cyan dashed line) lacking both His-GFP (green nuclei, B) and Ubi-RFP (magenta cytoplasm) possess similar levels of mitochondrial ATP5A puncta (red) that is stained by antibody compared to either $Atg8^{\Delta}$ single mutant (magenta positive and green negative, yellow dashed line, A) or $Atg9^{D51}$ single mutant cells (magenta negative and green positive, white dashed line, A). The double mutant and single mutant cell clones were induced in the same intestines.

(F) Quantification of ATP5A puncta in $Atg8^{\Delta}$ single mutant, $Atg9^{D51}$ single mutant and $Atg8^{\Delta}$ $Atg9^{D51}$ double mutant cells compared to neighboring control cells. n = 29 (Ctrl), n = 13 (*Atg8a*), n = 12 (*Atg9*), n = 11 (*Atg8 Atg9*) cells were measured.

(G) Diagram of ss-pHluorin-mKate2-KDEL-V5 sensor.

(H) Intestines that express ss-pHluorin-mKate2-KDEL-V5 (ss-PK-KDEL) in all enterocytes exhibit increased cell size and decreased ss-mKate2-KDEL (red puncta, mK2) and LysoTracker (gray) puncta in the larger Lsn^{SS6} mutant enterocytes (white dotted line) compared to smaller neighboring control cells which possess co-localization of both ss-mKate2-KDEL and LysoTracker (gray) puncta, and ss-mKate2-KDEL and ss-pHluorin-KDEL (green) puncta compared to neighboring control cells.

(I) Intestines that express ss-pHluorin-mKate2-KDEL-V5 (ss-PK-KDEL) in all cells exhibit decreased ss-mKate2-KDEL puncta (red puncta, mK2) co-localization with LysoTracker (gray) in the larger $Atg16^{\Delta}$ mutant enterocytes (white dotted line) compared to smaller neighboring control cells.

(J) Intestines that express ss-pHluorin-mKate2-KDEL-V5 (ss-PK-KDEL) in all enterocytes exhibit decreased ss-mKate2-KDEL (red puncta, mK2) and LysoTracker (gray) puncta in the larger Lsn^{SS6} and $Atg16^{\Delta}$ double mutant enterocytes (white dotted line) compared to smaller neighboring control cells which possess co-localization of both ss-mKate2-KDEL and LysoTracker (gray) puncta, and ss-mKate2-KDEL and ss-pHluorin-KDEL (green) puncta compared to neighboring control cells.

(K) Quantification of the ratio of ss-mKate2-KDEL puncta co-localized with LysoTracker of total ss-mKate2-KDEL puncta in *Lsn* single mutant (E), *Atg16* single mutant (F), and *Lsn* and *Atg16* double mutant enterocytes (G) normalized to

neighboring control cells in each genotype. n = 18 (Ctrl), n = 5 (*Lsn*), n = 5 (*Atg16*), and n = 8 (*Lsn, Atg16*) cells were measured.

All animals were staged 2 hours APF. Scale bars in (A-B) and related insets represent 20 µm. Scale bars in (H-J) insets represent 5 µm Scale bars in (D-E) and related insets represent 0.5 µm and 0.2 µm. All insets are from indicated rectangles (white rectangle = mutant cell, yellow rectangle = control cell). White arrows indicate co-localized ss-mKate2-KDEL and LysoTracker and yellow arrows indicate co-localized ss-pHluorin-KDEL and ss-mKate2-KDEL. Data are presented as mean ± SEM. n.s. = not significant, $^{**}p < 0.01$, $^{****}p < 0.0001$ from a two-tailed unpaired t-test and one-way ANOVA corrected by Tukey's post hoc test. Each data point represents one mutant cell/neighboring cell. Representative of 3 or more independent biological experiments from ⩾3 different animals.

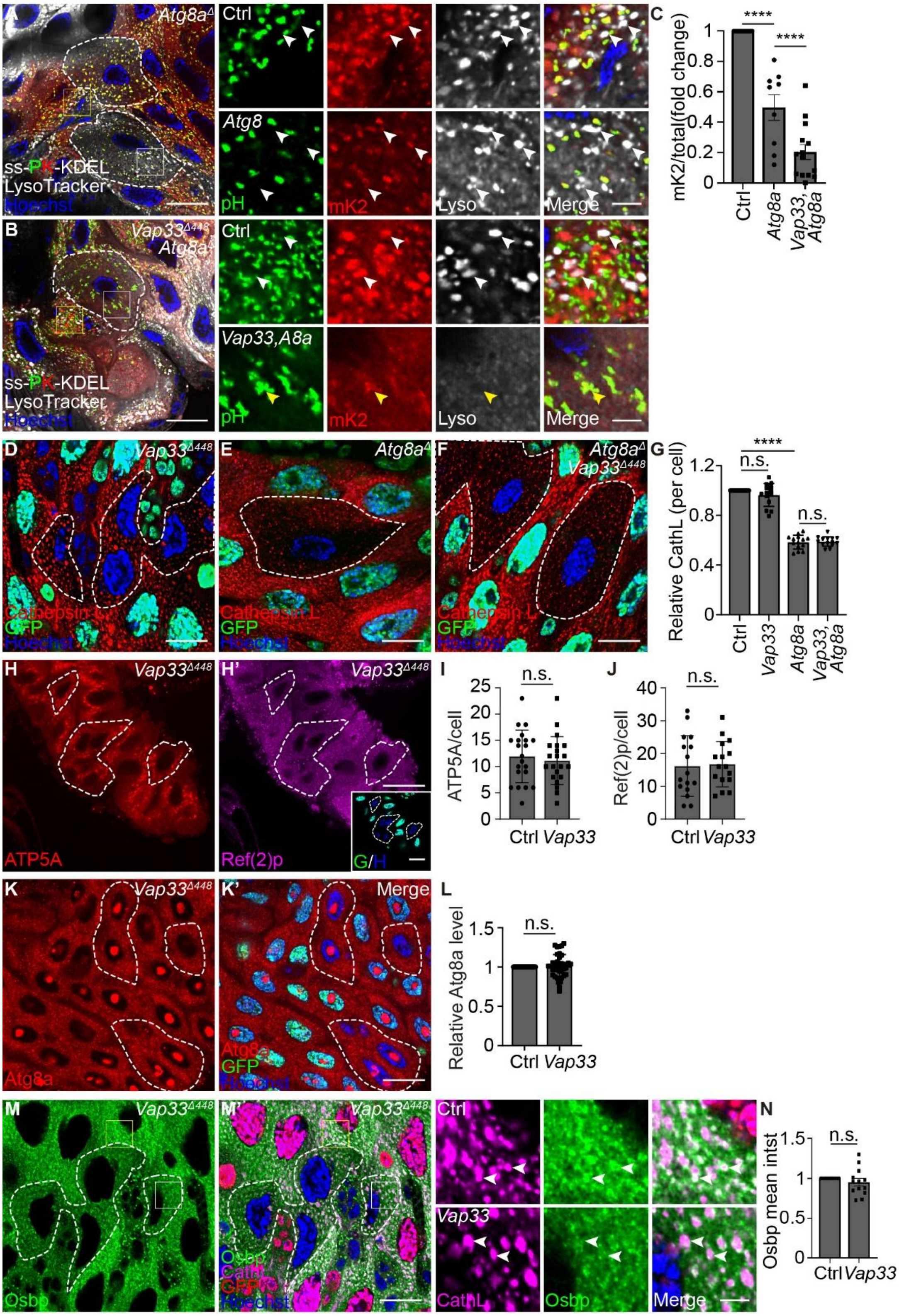

Figure S2. Vap33 is required for Atg8a-independent lysosomal ER clearance.

Related to Figure 2.

(A) Intestines that express ss-pHluorin-mKate2-KDEL-V5 (ss-PK-KDEL) in all cells exhibit increased cell size and decreased but not absent ss-mKate2-KDEL puncta (red puncta, mK2) co-localization with LysoTracker (gray) in *Atg8a*$^{\Delta}$ mutant enterocytes (white dotted line) compared to smaller neighboring control cells.

(B) Intestines that express ss-pHluorin-mKate2-KDEL-V5 (ss-PK-KDEL) in all cells exhibit increased cell size, decreased co-localization of ss-mKate2-KDEL and LysoTracker (gray) in *Vap33*$^{\Delta 448}$ *Atg8*$^{\Delta}$ double mutant enterocytes (white dotted line) compared to *Atg8a* single mutant cells (A).

(C) Quantification of the ratio of ss-mKate2-KDEL puncta co-localized with LysoTracker of total ss-mKate2-KDEL puncta in *Atg8a* single mutant enterocytes (A) and *Vap33* and *Atg8a* double mutant enterocytes (B) normalized to neighboring control cells of each genotype. n = 24 (Ctrl), n = 10 (*Atg8a*), n = 14 (*Vap33*, *Atg8a*) cells were measured.

(D) *Vap33*$^{\Delta 448}$ mutant enterocyte cells (white dotted line, non-green) exhibit similar Cathepsin L (red) puncta compared to neighboring control cells (green).

(E) *Atg8a*$^{\Delta}$ mutant enterocyte cells (white dotted line, non-green) exhibit decreased Cathepsin L (red) puncta compared to neighboring control cells (green).

(F) *Atg8a*$^{\Delta}$ *Vap33* double mutant enterocyte cells (white dotted line, non-green) exhibit similar decreased Cathepsin L (red) puncta compared to *Atg8a* single mutant cells (E).

(G) Quantification of Cathepsin L puncta per cell in single and double mutant enterocytes compared to neighboring control cells. n = 14 (Ctrl), n = 14 (*Vap33*), n = 14 (*Atg8a*), and n = 14 (*Vap33 Atg8a*) cells were quantified.

(H and H') *Vap33*$^{\Delta 448}$ mutant enterocytes (white dotted line, non-green in the nuclei) possess similar ATP5A and Ref(2)p puncta compared to neighboring control cells. ATP5A and Ref(2)p were immunolabeled using antibodies.

(I) Quantification of ATP5A puncta of *Vap33* mutant compared to control cells. n = 21 (Ctrl), n = 21 (*Vap33*) cells were measured.

(J) Quantification of Ref(2)p puncta of *Vap33* mutant compared to control cells. n = 16 (Ctrl), n = 16 (*Vap33*) cells were measured.

(K and K') *Vap33*$^{\Delta 448}$ mutant enterocytes (white dotted line, non-green in the nuclei) possess similar Atg8a puncta compared to neighboring control cells. Atg8a was immunolabeled by antibody.

(L) Quantification of Atg8a intensity of *Vap33* mutant normalized to control cells. n = 38 (Ctrl), n = 38 (*Vap33*) cells were measured.

(M and M') Intestines that express V5-3×FLAG-Osbp in all cells exhibit similar Osbp intensity in *Vap33*$^{\Delta 448}$ mutant enterocytes lacking RFP (white dotted line) compared to neighboring control cells.

(N) Quantification of Osbp mean intensity in *Vap33* mutant enterocytes compared to neighboring control cells. n = 13 (Ctrl) and n = 13 (*Vap33*) cells were measured.

All animals were staged 2 hours APF. Scale bars in (A), (B), (D-F), (H'), (K') and (M') represent 20 μm, and (A) (B) and (M) insets represent 5 μm. Insets in (A), (B) and (M) are from indicated rectangles (white rectangle = mutant cell, yellow rectangle = control cell). White arrows in (A-B) indicate co-localized ss-mKate2-KDEL and LysoTracker and yellow arrows in (A-B) indicate co-localized ss-pHluorin-KDEL and ss-mKate2-KDEL. White arrows in (M) insets indicate co-localized Osbp and Cathepsin L puncta. Data are presented as mean ± SEM. n.s. = not significant, ****p < 0.0001 from one-way ANOVA corrected by Tukey's post hoc test and unpaired, two-tailed t test. Each data point represents one mutant cell/neighboring cell. Representative of 3 or more independent biological experiments from ⩾3 different animals.

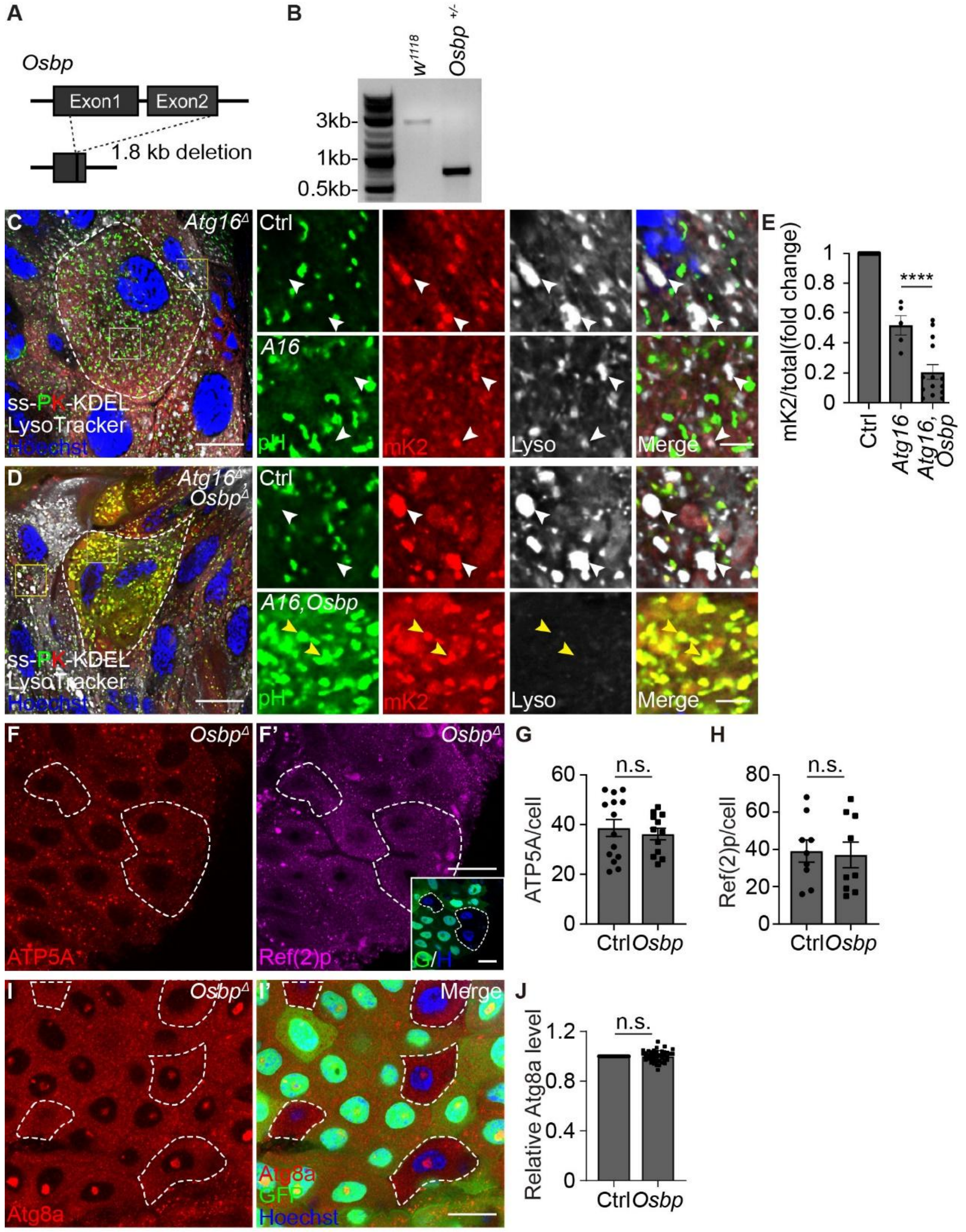

Figure S3. Osbp is required for macroautophagy-independent lysosomal ER clearance. Related to Figure 3.

(A) Diagram of *Osbp* deletion.

(B) Genotyping of heterozygous *Osbp* and control w^{1118} flies.

(C) Intestines that express ss-pHluorin-mKate2-KDEL-V5 (ss-PK-KDEL) in all cells exhibit increased cell size and decreased ss-mKate2-KDEL puncta (red puncta, mK2) co-localization with LysoTracker (gray) in *Atg16*$^{\Delta}$ mutant enterocytes (white dotted line) compared to smaller neighboring control cells.

(D) Intestines that express ss-pHluorin-mKate2-KDEL-V5 (ss-PK-KDEL) in all cells exhibit decreased ss-mKate2-KDEL puncta (red puncta, mK2) co-localization with LysoTracker (gray) in larger *Atg16*$^{\Delta}$ and *Osbp*$^{\Delta}$ double mutant enterocytes (white dotted line) compared to smaller neighboring control cells.

(E) Quantification of the ratio of ss-mKate2-KDEL puncta co-localized with LysoTracker of total ss-mKate2-KDEL puncta in *Atg16* single mutant enterocytes (C), and *Atg16* and *Osbp* (D) double mutant enterocytes normalized to neighboring control cells in each genotype. n = 19 (Ctrl), n = 5 (*Atg16*) and n = 14 (*Atg16*, *Osbp*) cells were measured.

(F and F') *Osbp*$^{\Delta}$ mutant enterocyte cells (white dotted lines, cells lacking nuclear GFP) possess similar ATP5A (red) and Ref(2)p (magenta) puncta compared to neighboring control cells (green). ATP5A and Ref(2)p were immunolabeled using antibodies.

(G) Quantification of ATP5A puncta in *Osbp* mutant and control cells. n = 14 (Ctrl), n = 12 (*Osbp*) cells were measured.

(H) Quantification of Ref(2)p puncta in *Osbp* mutant and control cells. n = 9 (Ctrl), n = 9 (*Osbp*) cells were measured.

(I and I') *Osbp*$^{\Delta}$ mutant enterocyte cells (white dotted lines, cells lacking GFP) possess similar Atg8a (red) puncta compared to neighboring control cells (green). Atg8a was immunolabeled by antibody.

(J) Quantification of Atg8a puncta in *Osbp* mutant and control cells. n = 43 (Ctrl), n = 43 (*Osbp*) cells were measured.

All animals were staged 2 hours APF. Scale bars in (C), (D), (F') and related inset and (I') represent 20 μm, and scale bars in (C) and (D) insets represent 5 μm. All insets are from indicated rectangles (white rectangle = mutant cell, yellow rectangle = control cell). White arrows in (C) and (D) indicate co-localized ss-mKate2-KDEL and LysoTracker and yellow arrows in (D) indicate co-localized ss-pHluorin-KDEL and ss-mKate2-KDEL. Data are presented as mean ± SEM. ***$p < 0.001$ from one-way ANOVA corrected by Tukey's post hoc. Each data point represents one mutant cell/neighboring cell. Representative of 3 or more independent biological experiments from ≥3 different animals.

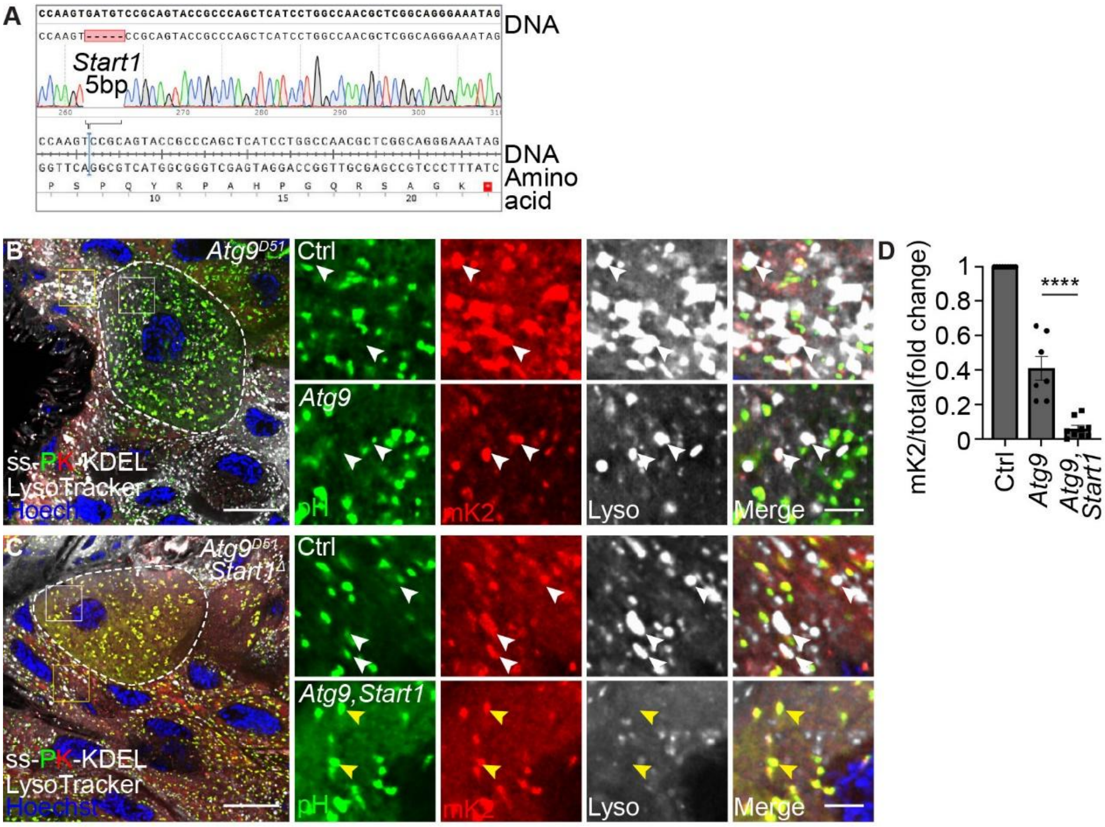

Figure S4. Start1 is required for macroautophagy-independent lysosomal ER clearance. Related to Figure 4.

(A) Diagram of the *Start1* (*Start1*$^{\Delta}$) mutant allele with a 5 bp deletion in exon 1 creating a frameshift and an early stop codon 44 bp downstream of the deletion.

(B) Intestines that express ss-pHluorin-mKate2-KDEL-V5 (ss-PK-KDEL) in all cells exhibit decreased ss-mKate2-KDEL puncta (red puncta, mK2) co-localization with LysoTracker (gray) in the larger *Atg9*^{D51} mutant larger enterocytes (white dotted line) compared to smaller neighboring control cells.

(C) Intestines that express ss-pHluorin-mKate2-KDEL-V5 (ss-PK-KDEL) in all cells exhibit decreased ss-mKate2-KDEL puncta (red puncta, mK2) co-localization with LysoTracker (gray) in the larger *Atg9*^{D51} and *Start1*$^{\Delta}$ double mutant enterocytes (white dotted line) compared to smaller neighboring control cells.

(D) Quantification of the ratio of ss-mKate2-KDEL puncta co-localized with LysoTracker of total ss-mKate2-KDEL puncta in *Atg9* single mutant enterocytes (B) and *Atg9* and *Start1* (C) double mutant enterocytes normalized to neighboring control cells in each genotype. n = 16 (Ctrl), n = 7 (*Atg9*), and n = 9 (*Atg9*, *Start1*) cells were measured.

All animals were staged 2 hours APF. Scale bars in (B-C) represent 20 μm, and scale bars in all insets represent 5 μm. All insets are from indicated rectangles (white

rectangle = mutant cell, yellow rectangle = control cell). White arrows in (B-C) indicate co-localized ss-mKate2-KDEL and LysoTracker and yellow arrows in (C) indicate co-localized ss-pHluorin-KDEL and ss-mKate2-KDEL. Each data point represents one mutant cell/neighboring cell. Representative of 3 or more independent biological experiments from ⩾3 different animals.

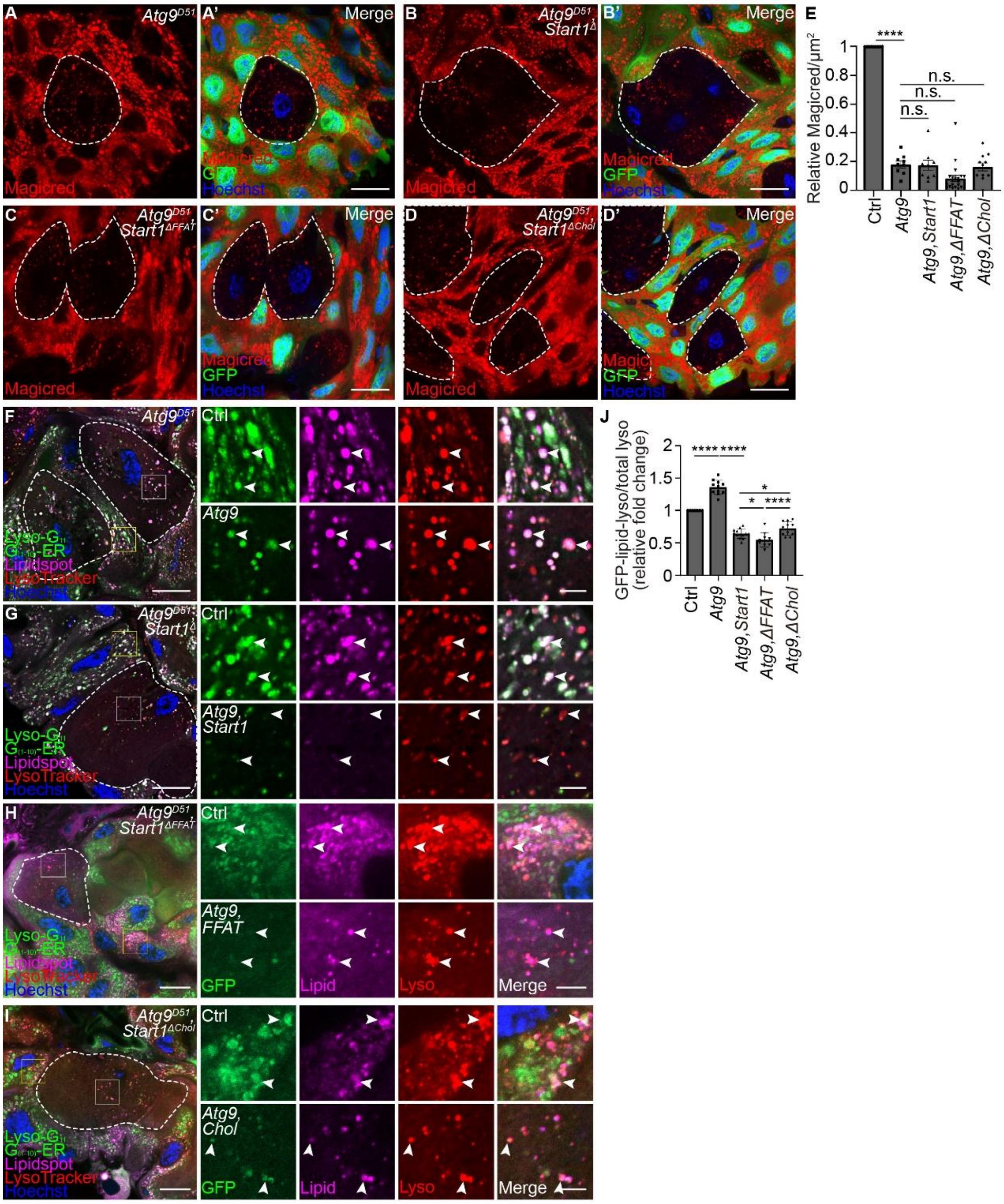

Figure S5. Start1 regulates lysosome and ER contact but not lysosome function. Related to Figure 5.

(A, A') *Atg9*^{D51},*Start1*^{Δ} (B, B'), *Atg9*^{D51},*Start1*^{ΔFFAT} (C, C') and *Atg9*^{D51},*Start1*^{ΔChol} (D, D') double mutant enterocyte cells possess similar Magic Red (red) puncta compared to *Atg9*^{D51} (A, A') single mutant cells. All single and double mutant cells possess decreased Magic Red (red) puncta compared to relative neighboring control cells. All mutant cells are lack of GFP expression (non-green), and control cells express GFP (green).

(E) Quantification of Magic Red puncta/μm^2 in *Atg9^{D51}* (A) single mutant cells *Atg9^{D51}Start1$^{\Delta}$* (B), *Atg9^{D51}Start1$^{\Delta FFAT}$* (C) and *Atg9^{D51}Start1$^{\Delta Chol}$* (D) double mutant enterocyte cells normalized to neighboring control cells in each genotype. n = 49 (Ctrl), n = 9 (*Atg9*), n = 9 (*Atg9*, *Start1*), n = 21 (*Atg9*, *△FFAT*), and n = 10 (*Atg9*, *△Chol*) cells were measured.

(F-I) *Atg9^{D51}Start1$^{\Delta}$* (G), *Atg9^{D51}Start1$^{\Delta FFAT}$* (H) and *Atg9^{D51}Start1$^{\Delta Chol}$* (I) double mutant enterocytes (white dotted line, larger cell compared to neighboring control cells) that express Lyso-GFP_{11}×7-V5-P2A-FLAG-$GFP_{(1\text{-}10)}$-ER (Lyso-G_{11}-$G_{(1\text{-}10)}$-ER) in all cells exhibit decreased GFP co-localization with Lipidspot (magenta) and LysoTracker (red) puncta compared to *Atg9^{D51}* single mutant enterocytes (F, white dotted line, larger cell compared to neighboring control cells).

(J) Quantification of the ratio of co-localized GFP, Lipidspot, and LysoTracker puncta (GFP-lipid-lyso) of total LysoTracker puncta (total lyso) in enterocytes of each mutant genotype normalized to neighboring control cells. n = 45 (Ctrl), n = 11 (*Atg9*), n = 13 (*Atg9*, *Start1*), n = 10 (*Atg9*, *△FFAT*), and n = 11 (*Atg9*, *△Chol*) cells were measured. All animals were staged 2 hours APF.

Scale bars in (A-D), (F-I) represent 20 μm, and scale bars in all insets represent 5 μm. All insets are from indicated rectangles (white rectangle = mutant cell, yellow rectangle = control cell). White arrows in (F-I) indicate co-localized GFP, Lipidspot and LysoTracker puncta. Data are presented as mean ± SEM. n.s. = not significant, *p < 0.05, ****p < 0.0001 from Fisher's LSD test. Each data point represents one mutant cell/neighboring cell. Representative of 3 or more independent biological experiments from ⩾3 different animals.

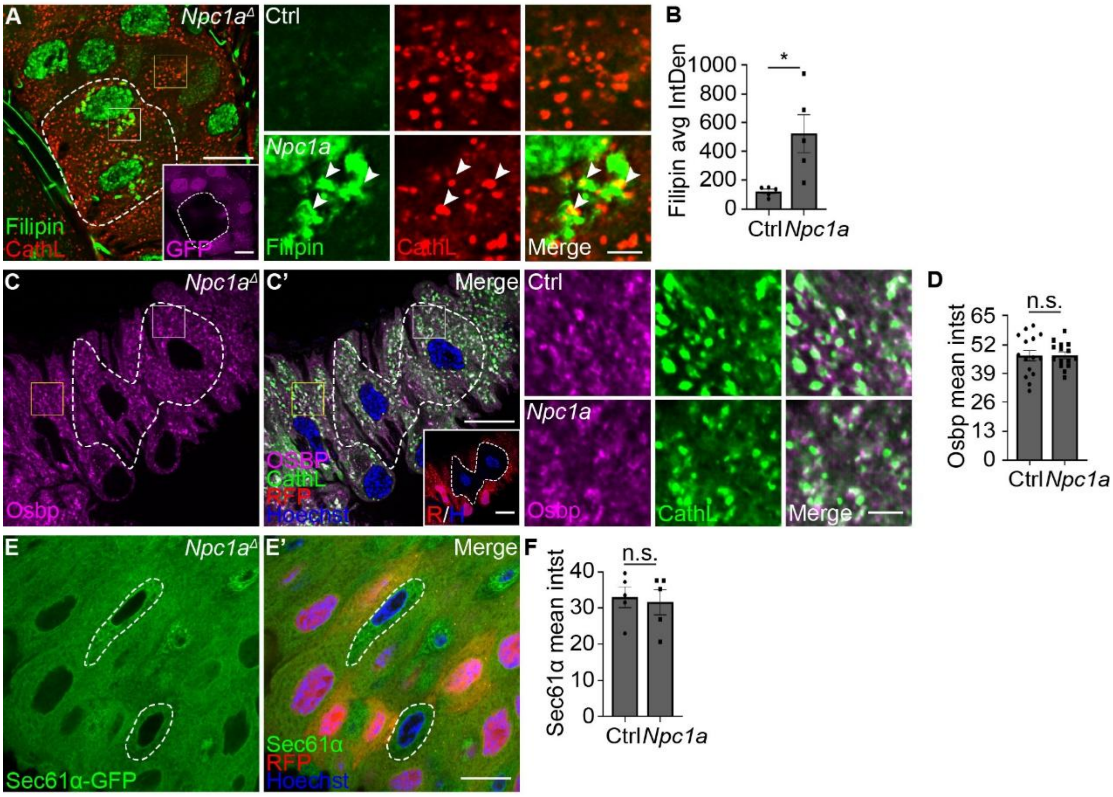

Figure S6. Npc1a is not required for ER clearance. Related to Figure 6.

(A) *Npc1a*$^{\Delta}$ loss-of-function mutant enterocytes lacking GFP (magenta, white dotted line) possess increased density of large Filipin puncta (green) that partially co-localized with Cathepsin L puncta (red, CathL) compared to neighboring control cells which exhibit less Filipin puncta.

(B) Quantification of average integrated density (IntDen) of each Filipin puncta in *Npc1a* mutant enterocytes compared to control cells. n = 5 (Ctrl) and n = 5 (*Npc1*) cells were measured.

(C and C’) Intestines expressing V5-3×FLAG-Osbp (magenta) in all cells exhibit similar intensity of Osbp co-localization with Cathepsin L (CathL, green) puncta in *Npc1a*$^{\Delta}$ mutant enterocytes lacking RFP (red, white dotted line) compared to neighboring control cells.

(D) Quantification of Osbp mean intensity (mean intst) of *Npc1a* mutant enterocytes compared to control cells. n = 15 (Ctrl) and n = 15 (*Npc1a*) cells were measured.

(E and E’) Intestines expressing Sec61α-GFP (green) in all cells exhibit similar intensity of Sec61α-GFP in *Npc1a*$^{\Delta}$ mutant enterocytes lacking RFP (red, white dotted line) compared to neighboring control cells.

(F) Quantification of mean intensity of Sec61α-GFP in *Npc1a*$^{\Delta}$ mutant enterocytes compared to control cells. n = 5 (Ctrl) and n = 5 (*Npc1a*) cells were measured.

All animals were staged 2 hours APF. Scale bars in (a and inset at the right bottom corner), (C' and inset at the right bottom corner) and (E') represent 20 μm, and scale bars in (A) and (C) insets represent 5 μm. Insets are from indicated rectangles (white rectangle = mutant cell, yellow rectangle = control cell). White arrows in (A) insets indicate co-localized Filipin and Cathepsin L puncta. Data are presented as mean ± SEM. n.s. = not significant, *$p < 0.05$ from unpaired, two-tailed t test. Each data point represents one mutant cell/neighboring cell. Representative of 3 or more independent biological experiments from ⩾3 different animals.
