# Supplementary material for: Cholesterol transfer proteins promote Atg-independent ER clearance by lysosomes
Source: Cell Rep. Author manuscript; Available in PMC 2026 Jul 13. (PMC13358760; doi:10.1016/j.celrep.2026.117537)
Supplement: 2 [file NIHMS2190755-supplement-2.docx]

**SUPPLEMENTAL TABLE 1. Oligonucleotides and gBlocks.**

| REAGENTS or RESOURCE | SOURCE | IDENTIFIER |
| --- | --- | --- |
| Oligonucleotides (5’-3’) | | |
| Forward primer to screen *Osbp^Δ^*  GCTATTCGCAGTCTCGAAGC | IDT | N/A |
| Reverse primer to screen *Osbp^Δ^*  TGGCCTCCCAGTAAGTGTTC | IDT | N/A |
| Forward primer to screen *Start1^Δ^*  AGTCATTTGTGCGTATCGGC | IDT | N/A |
| Reverse primer to screen *Start1^Δ^*  TCGGTGGGAGATCGTTATGG | IDT | N/A |
| Forward primer to screen V5-3xFLAG-Vap33  CAAACAGCCAGCAAGAACAA | IDT | N/A |
| Reverse primer to screen V5-3xFLAG-Vap33  GACCCCGAAAAGAACAACAA | IDT | N/A |
| Forward primer to screen V5-3xFLAG-OSBP  CAATCGCCAGTCATCGTACG | IDT | N/A |
| Reverse primer to screen V5-3xFLAG-OSBP  CCAGCCTTTCATCTCCGGTA | IDT | N/A |
| V5-3xFLAG-Vap33 gRNA1 sense oligo  GTCGGCAAGAAATGGGAGGTGAAT | IDT | N/A |
| V5-3xFLAG-Vap33 gRNA1 antisense oligo  AAACATTCACCTCCCATTTCTTGC | IDT | N/A |
| V5-3xFLAG-Vap33 gRNA2 sense oligo  GTCGGAGTGATTTGCTCATTGTGG | IDT | N/A |
| V5-3xFLAG-Vap33 gRNA2 antisense oligo  AAACCCACAATGAGCAAATCACTC | IDT | N/A |
| V5-3xFLAG-Osbp gRNA1 sense oligo  GTCGGTAGTAGCTCAGCACGCCCT | IDT | N/A |
| V5-3xFLAG-Osbp gRNA1 antisense oligo  AAACAGGGCGTGCTGAGCTACTAC | IDT | N/A |
| V5-3xFLAG-Osbp gRNA2 sense oligo  GTCGGCCAGCGGCGTCTGTCATGA | IDT | N/A |
| V5-3xFLAG-Osbp gRNA2 antisense oligo  AAACTCATGACAGACGCCGCTGGC | IDT | N/A |
| *Start1ΔFFAT* gRNA1 sense oligo  CTTCGTCCATGAGTGAATAAAAAT | IDT | N/A |
| *Start1ΔFFAT* gRNA1 antisense oligo  AAACATTTTTATTCACTCATGGAC | IDT | N/A |
| *Start1ΔFFAT* gRNA2 sense oligo  CTTCGATCAGACTCTTTGGAGATA | IDT | N/A |
| *Start1ΔFFAT* gRNA2 antisense oligo  AAACTATCTCCAAAGAGTCTGATC | IDT | N/A |
| *Start1ΔChol* gRNA1 sense oligo  CTTCGTAAGGGTAAAGCAATGATC | IDT | N/A |
| *Start1ΔChol* gRNA1 antisense oligo  AAACGATCATTGCTTTACCCTTAC | IDT | N/A |
| *Start1ΔChol* gRNA2 sense oligo  CTTCGCGAAACACTCAATATAATC | IDT | N/A |
| *Start1ΔChol* gRNA2 antisense oligo  AAACGATTATATTGAGTGTTTCGC | IDT | N/A |
| Forward primer to screen *Start1 ΔFFAT*  TGCCTTCCACAAACAAATCGTG | IDT | N/A |
| Reverse primer to screen *Start1 ΔFFAT*  CCCACTCACGCGTACTATCT | IDT | N/A |
| Forward primer to screen *Start1 Δchol*  GGAACAGCCTGCCATTTTGA | IDT | N/A |
| Reverse primer to screen *Start1 Δchol*  AGTCCCGAAACCAACCTTGTG | IDT | N/A |
| *Osbp* gRNA1 TCCGTATGCGTTCCTCCGGCCGG (provided by flybase) | Bloomington *Drosophila* stock center (BDSC) | 92577 |
| *Osbp* gRNA2 ATATGGCCTACCCTCTGCGGCGG (provided by flybase) | Bloomington *Drosophila* stock center (BDSC) | 92577 |
| *Start1* gRNA GCGGTACTGCGGACATCACTTGG (provided by flybase) | Bloomington *Drosophila* stock center (BDSC) | 82816 |
| *Vap33* gRNA ACGTACGCAGTAGCGTTTCGGGG (provided by flybase) | Bloomington *Drosophila* stock center (BDSC) | 81751 |
| V5-3xFLAG-Vap33 homology directed repair (HDR) template gblock  TTTCAACTGAAGTTTGCGAAGAAACCGAAGCGTGGTAAACCACTGAAATCGAAAATATCGACAGAAAAGCGACCTAAAGTCGGTGAAGAAGTCGCACGTTGATCGTTGTGTTTTTTTCCCGAAATTTTCTGCAAAAAGCCCGTGCGTGCGTGAGTTTCTCTGGCTCTTGCTTTTTTTTTGTCCATGCGTGTGTGTGTGGTCGCATAAATTTACCGATATTTCGCCTGTGAGAGCGAAACGAACGAAAAACGAAAGAAAAAAAGAGAGACGAGTAAAGTAAAACGAAACAGGCATAAAAACAGCAGCAGTTTTCTTGATATATTTGGCTAAAAAACGCAAACGTAAGCGAAAAATTTGAAATCCCTCTTTTAATTTAGTTGTGTGCCTGTGTCTCGTATCTATGCGTGCGTCTGCATTTTAATATAATTTTGTGTGCATTTTCTTAACAGCAAACAGCCAGCAAGAACAACAAATAGCTGGGCAAAAACAGGACGCACAAAAAATAAAATTAAAACGATAAGAGGCGAAAAGCGGAGAGAGTGAAATTCTCGGCAGCAACAACGACAAGAACAACACCAGGAGCAGCAGCAACAACAACAACAAAAGCCAGCCGCCACAATGGACTACAAAGACCATGACGGTGATTATAAAGATCATGACATCGATTACAAGGATGACGATGACAAGGGTAAGCCTATCCCTAACCCTCTCCTCGGTCTCGATTCTACGGGTGGAGGAGGTTCCGGACTCAGATCTTCTAGAGGGCCCTTCGAAAGCAAATCACTCTTTGATCTTCCGTTGACCATTGAACCAGAACATGAGTTGCGTTTTGTGGGTAAGTTTATTTTACATTATTTTCGCGTTTTTGCGGAAAGGTCGCTGCATATCGACCAAAAAAGCAGATTCACCTCCCATTTCTTGCAAAGAGCAAACTGCAAGTTTCACTTTTTCTCTCTCTCACGCGCACATTTAATCTCCCTCCCTCTACGCTTTCTATTGCCATCTCGCTCGATTGGCTATCAATAATTTCCGTTGTTTTTGTTCTTCCTTTACATACGACACCTTTTATTGTTGTTCTTTTCGGGGTCTACGTGTATCATCGCTCTGCTTCTTTCCGTTTTTTTTCTTGTTTTTTTTTTTGCTTCAGCTCTTTAATGTATTTTATTTTTTGATGCCGGCAGCTGGCGTCGCTCTCTTTCTCACTTGCACTCGCCATCCCTCTCCCTCTTGCTCACTCGTTTCGCGTTTTTGGCTAGCATTATATTTTTATACGCAAAAACTTGTCATGGGCCATTTTAATTTTTTCTTGTCTTTTGGCTCCTTGAATTTTGGCCAAAAAGCAACAGCAGCAGTGACAAAAACAATGCAAAGAGCTCGCACAGTGGGTGCGACAAGTGGCACGGTTGTCATCGCATTGTTATTGAACTCCTGTTGCCAAGCTGCATTTTCCAGTGCATAAACATTTAATTTTTTTCGCGAAATTTTATCACAGTCTCAATTGCAGAACGAAATGTCTACTTAAATGACCAATCATTGTTGATTGCGATCTAGAATAATACACCAATAACCATTCACCATTTGTATTTTGTCTTATTGAAAAAAATTGAGTTAACTTTTCAAGTATTTTGCTATCGCTTCCATTGTGCGCAAATATTTTAATATCTGTGTATGTGTGAGTGAGCCTTAGCCCCATTCTTTTGCAACCCCCTCGCCCACGTTTCCCGTTAGATCGTGAATAGTGCGGTCGGCG | IDT | N/A |
| V5-3xFLAG-Osbp homology directed repair (HDR) template gblock  AAGCTTTCTAACGGCACAGTGTTTGAAAAAGATTTTTTCAGATCGCAGATTATCGTTGCCTATCGCCCATCGATATGTGTAGGAGGAAACGCAACCAACCACCTGGTAACACTGACACTCACACACCTGCAGATGCGCCAGCACACACACACAGAACACACACGCACACAGACGCGCACTGAACTTCCACTACAGTTTCTTTATTTTCTGCAGGAAAATTAGAAGCAAAACGACACGAAATTCGCAGTGAACTCGCGGCGGACTCGCGACCGTAACGTAATCGGAATCTCGGTGGTGATCCGGAGGCCAAGTGCACACCGTGCGATGCTAGCCACTGGCCAGCAATCAGCCGTCGCCGTCCAATAACCAATAGCATTCGAATTTCAAGTCGGAGTTGCGGAACGGAACCAATCCGACCAATCGCCAGTCATCGTACGTGGCGCTATTCGCAGTCTCGAAGCAAGTGCTTCATCCGGCCGGAGGAACGCATACGGATACCGGAACCGGTTGCCATCATGGACTACAAAGACCATGACGGTGATTATAAAGATCATGACATCGATTACAAGGATGACGATGACAAGGGTAAGCCTATCCCTAACCCTCTCCTCGGTCTCGATTCTACGGGTGGAGGAGGTTCCGGACTCAGATCTTCTAGAGGGCCCTTCGAAACAGACGCCGCTGGCAATGCGCTGGCCGAGAAGGGCCTACCGGAGATGAAAGGCTGGCTGCTAAAGTGGACCAATTACATCAAGGGCTACCAGCGGCGATGGTTCGTGCTATCAAAGGGCGTGCTGAGCTACTACCGCAACCAGTCGGAGATTAACCACACGTGCCGGGGCACCATATCGCTTCACGGAGCCCTCATCCACACGGTCGATTCGTGCACGTTCGTAATCTCGAACGGCGGCACCCAAACGTTCCACATCAAGGCCGGCACCGAGGTGGAGCGCCAGTCGTGGGTCACCGCCCTGGAGCTGGCAAAGGCTAAGGCAATCCGGGCCATCGAATGCGAGGAGGAGGAGGAGACGGAAACGGCACATGTGGTGCCCAGCCAGGAGATCAGCTCGGTGGTCCGAGATCTCACCGATCGGCTGGAGAGCATGCGTACCTGCTACGACCTGATCACCAAGCATGGCGCAGCCTTGCAGCGCGCTCTCAACGATCTGGAGACGAACGAGGAGGAGTCGCTGGGCAGCCGCACGAAGATCGTCAACGAGAGGGCGACCCTCTTCCGGATAACCTCCAATGCGATGATCAACGCCGGCAACGACTACCTGCACACGGCAGAGGCACAAGGCCATAA | IDT | N/A |
| *Start1ΔFFAT* homology directed repair (HDR) template gBlock  TGCCGATTTTTGGTGCTCATATTTTTCTACGCCATATTGTACATCAATCACTGGTCCATCATAGCGGTATAGTTTTGCTTTCCAATTATAGAATTCCTTTTATATCTAATTATTTCTGCAGCTCTCTACAAGTGGGTCTTGCTTGTTCCTCATCTCGAAGGTGTTTGTGTTCGATGTAAGCCTGAGAAACCAGCCACCTTAATGTACTCTTAGATAATTTCGTGTGAATCTTTCAGTGGCTGGATTCAAAGCAGCAGGTATTTGAGGTAATCCTCATAATAACCTCGTTCATACTGGCTTGGGGAGAAGCCTGGTTCCTGGACTGTAGGGTGATTCCTCAAGAGCGACATGCCCAACACTATTTCCGGAGTAAGTTACGCAGACAGATCCCCATATCATATTCATTGATTTTCGCATTTAAGCTATGACTTCAAATGATCGCACACCCATGGAACAGCCTGCCATTTTGATTGAGCAAGAACGGCCTCCGCAAAGTGTAACAGATGCAGCTTCACTCATGGACACGGCGCGCCATTCCGACGAGGAGGATGAGTTGGTATGTTATTCATGAATGCGCATTTTTAAGTCTGTCCAGAATAAGAAAGTGAATCTAGTGGAAATATAAAATGAAGCACAATTGATTTTGTCACAATATCTCCAAAGAGTCTGATCCAATGACATCGGTAGTTCTGGGAGTTTTTCCCGCATTTCTAAGATTTTTTATTCGTTCACTTTACAGGATGATGAGTACACACAAATGGGATTGGATTGCCTTCGAAAGGCCTACGAGATCATCGAGTCAAGTGACTGGAAGGTGGAAAAAGTTAACCAGAAAGGCGACACCATACACAGCACTCAGCGCGACAAGATTGGAAAGATCTACAAGTTGACGGTAAGGGTAAAGCAATGATCAGGGAATATTTCTTCTTATACGTAAACTTCACAGGCCCGCATCAAGTATCCTGCAAAGGCTCTGATGGAAGATCTGTTCTATCGCATTGAAGACTGTCCCAAGTGGAATCCTGCTCTTTTGGAGTCCAAGATAGTACGCGTGAGTGGGCACACAACATGACGATTTAAAACTCCTGATTATATTGAGTGTTTCGCACAGAAAATCAACTCCTACACCGATATTACCTATCAGGTATCCGTGGGCGGAGGAGGTGGCATGG | IDT | N/A |
| *Start1ΔChol* homology directed repair (HDR) template gBlock  ATATCATATTCATTGATTTTCGCATTTAAGCTATGACTTCAAATGATCGCACACCCATGGAACAGCCTGCCATTTTGATTGAGCAAGAACGGCCTCCGCAAAGTGTAACCGATTTTTATTCACTCATGGACACGGCTCGTCATTCCGACGAGGAGGATGAGTTGGTATGTTATTCATGAATGCGCATTTTTAAGTCTGTCCAGAATAAGAAAGTGAATCTAGTGGAAATATAAAATGAAGCACAATTGATTTTGTCACCATATCTCCAAAGAGTCTGATCCAATGACATCGGTAGTTCTGGGAGTTTTTCCCGCATTTCTAAGATTTTTTATTCGTTCACTTTACAGGATGATGAGTACACACAAATGGGATTGGATTGCCTTCGAAAGGCCTACGAGATCATCGAGTCAAGTGACTGGAAGGTGGAAAAAGTTAACCAGAAAGGCGACACCATACACAGCACTCAGCGCGACAAGATTGGAAAGATCTACAAGTTGACGGTAAGGGTAAAGCAATGATCAAGGAATATTTCTTCTTATACGTAAACTTCACAGGCCCGCATCAAGTATCCTGCAAAGGCTCTGATGGAAGATCTGTTCTATCGCATTGAAGACGCTCCCAAGGCGGATCCTGCTCTTTTGGAGTCCAAGATAGTACGCGTGAGTGGGCACACAACATGACGATTTAAAACTCATGATTATATTGAGTGTTTCGCACAGAAAATCAACTCCTACACCGATATTACCTATCAGGTATCCGTGGGCGGAGGAGGTGGCATGGTGAAGAGCCGCGACTTCGTGAACTTGCGGTCTTGTAGGCTCTTTTACAATGGTCAAATCTGCGATGACGATGAGACGGCTCAGCTCAGCAGCGATGATGGGAACAGCAGTCTAAATCGGTCTTGCGAGGGTAGTGTTAGTACCATTTCCGATGGTGACTCAAACACCCCACTGCTGCCCAGTAGCGTGTCTAGTTGCAAGGCAACGTTTCCCACTTCATCCAAGGGAGCCGCTATGCCTTTTGACACCCTGGGCAACAGCTTGGGCGCCAAGAGCCTAGGTCCCATCGTGAACTTTGACGAGGAGCCACCGCCATTGGATCAGGACGAGTTCGAGGATGCTAAGGACAAGGTCGACGGCGAAGCGAATAACATGACGAAACCAAATGTACCCAGCGTGGGAAAAACCAAGGACAGGGTTTGGGTCACTTCGGCGG | IDT | N/A |
| Lyso-GFP11x7-V5-P2A-FLAG-GFP(1-10)-ER gBlock  TGAATAGGGAATTGGAGATCTATGGAGCAACCAGCAACTGGAGGCGCCGCTGTCCAACCGGGCGTGTCCCCAGCGCACATCCACGACACGGATCAGCTCCTGGACCCCGTCCTCTTCTCCAACGACAATGGAAGCTACAAGCTAAATACAGTGCCCGCATTTAGCCTTCATCTGGTTATCTCCACTGCAATCTCCATTGTGGGGATCGTACTTGCAGCCTCGTTTCCAACCGATCGGCGCTGCGATGCATACTTTATAATGCTCTACCTACGGGCCACCTTCTGGGTCATCACATATCTCTTCGATCACTTCGTAAAGAAGCAACACGACAATCTACGCATGCAGGGCTATCACGACTTTCACCGCGAGACTAACATGCAGAAGGGGATTCCCCTTCAGCTGGTATCCCTGTGGAACTCCATGCTGCTTGCTGTCCAGGCCCTAATCCACCACTTCTATGCGGAGAACTTTTGGGAACATTGCGCCGCCGGGTGGCTGTCGCCGGTCAGCTATGTGACCGCCTTCACCGTGGCCGAGAATCTGGTGCTGGCCGTTTCCCACAGCTTATATATTGACAAGGTGCGTAAGTTCAACAGCGCCAAGCTGGCTCCGGATGTTTTGCGTGGTGCGGACCGCGCCGGCGGTTCCCTGGGCCTCATGCAACCAGGAGGCGACACAGAAGAGCTTCTGGAGAAGCAGGCCGACCTTATCGCCTATCTGCGTGACCATACACACAAGCTCAACCAGAAACTGCATCAAATGCAGACCAACGTGCGACCAGTGAGGGCACCACAAATTCCTGGTACCAAGGGCTCGGGCTCGACCTCGGGCTCGGGCACCGGCCGTGACCACATGGTCCTTCATGAGTATGTAAATGCTGCTGGGATTACAGGTGGCTCTGGAGGTAGAGATCATATGGTTCTCCACGAATACGTTAACGCCGCAGGCATCACTGGCGGTAGTGGAGGACGCGACCATATGGTACTACATGAATATGTCAATGCAGCCGGAATAACCGGAGGGTCCGGAGGCCGGGATCACATGGTGCTGCATGAGTATGTGAACGCGGCGGGTATAACTGGTGGGTCGGGCGGACGAGACCATATGGTGCTTCACGAATACGTAAACGCAGCTGGCATTACTGGCGGATCAGGTGGCAGGGATCACATGGTACTCCATGAGTACGTGAACGCTGCTGGAATCACAGGCGGTAGCGGCGGTCGGGACCATATGGTCCTGCACGAATATGTCAATGCTGCCGGTATCACCGGCAGTGGAAGCGGTGGTAAGCCTATCCCTAACCCTCTCCTCGGTCTCGATTCTACGGTCGACGCCACCAACTTCTCCCTGCTGAAGCAGGCCGGCGACGTGGAGGAGAACCCCGGCCCCGGATCCATGGACTACAAAGACCATGACGGTGATTATAAAGATCATGACATCGATTACAAGGATGACGATGACAAGGGTAGTGGGAGCGGTTCCAAAGGAGAAGAACTGTTTACCGGTGTTGTGCCAATTTTGGTTGAACTCGATGGTGATGTCAACGGACATAAGTTCTCAGTGAGAGGCGAAGGAGAAGGTGACGCCACCATTGGAAAATTGACTCTTAAATTCATCTGTACTACTGGTAAACTTCCTGTACCATGGCCGACTCTCGTAACAACGCTTACGTACGGAGTTCAGTGCTTTTCGAGATACCCAGACCATATGAAAAGACATGACTTTTTTAAGTCGGCTATGCCTGAAGGTTACGTGCAAGAAAGAACAATTTCGTTCAAAGATGATGGAAAATATAAAACTAGAGCAGTTGTTAAATTTGAAGGAGATACTTTGGTTAACCGCATTGAACTGAAAGGAACAGATTTTAAAGAAGATGGTAATATTCTTGGACACAAACTCGAATACAATTTTAATAGTCATAACGTATACATCACTGCTGATAAGCAAAAGAACGGAATTAAAGCGAATTTCACAGTACGCCATAATGTAGAAGATGGCAGTGTTCAACTTGCCGACCATTACCAACAAAACACCCCTATTGGAGACGGTCCGGTACTTCTTCCTGATAATCACTACCTCTCAACACAAACAGTCCTGAGCAAAGATCCAAATGAAAAATCTAGAAGTGCTGGAGGTAGTGCTGGTGGAAGTGCTGGCGGAAGTGCTGGAGGCAGTGCTGGTGGCCCTCGAGCTCAAGCTTCGAATTCGAGTGCTGGTGGTATCACCACCGTGGAGTCCAACTCCTCCTGGTGGACCAACTGGGTGATCCCCGCCATCTCCGCCCTGGTGGTGGCCCTGATGTACCGCCTGTACATGGCCGAGGACTAGGCGGCCGCGGATCTTTGTGAAGG | IDT | N/A |
| D4H*-GFP11x7-V5-P2A-FLAG-GFP(1-10)-ER gBlock  TGAATAGGGAATTGGAGATCTATGAAGGGAAAAATAAACTTAGATCATAGTGGAGCCTATGTTGCACAGTTTGAAGTAGCCTGGGATGAAGTTTCAGCCGACAAAGAAGGAAATGAAGTTTTAACTCATAAAACATGGGATGGAAATTATCAATCTAAAACAGCTCACTATTCAACAGTAATACCTCTTGAAGCTAATGCAAGAAATATAAGAATAAAAGCAAGAGAGTGTACAGGCCTTTGGTGGGAATGGTGGAGAGATGTTATAAGTGAATATGATGTTCCATTAACAAATAATATAAATGTTTCAATATGGGGAACAACTTTATACCCTGGATCTAGTATTACTTACAATGGTACCAAGGGCTCGGGCTCGACCTCGGGCTCGGGCACCGGCCGTGACCACATGGTCCTTCATGAGTATGTAAATGCTGCTGGGATTACAGGTGGCTCTGGAGGTAGAGATCATATGGTTCTCCACGAATACGTTAACGCCGCAGGCATCACTGGCGGTAGTGGAGGACGCGACCATATGGTACTACATGAATATGTCAATGCAGCCGGAATAACCGGAGGGTCCGGAGGCCGGGATCACATGGTGCTGCATGAGTATGTGAACGCGGCGGGTATAACTGGTGGGTCGGGCGGACGAGACCATATGGTGCTTCACGAATACGTAAACGCAGCTGGCATTACTGGCGGATCAGGTGGCAGGGATCACATGGTACTCCATGAGTACGTGAACGCTGCTGGAATCACAGGCGGTAGCGGCGGTCGGGACCATATGGTCCTGCACGAATATGTCAATGCTGCCGGTATCACCGGCAGTGGAAGCGGTGGTAAGCCTATCCCTAACCCTCTCCTCGGTCTCGATTCTACGGTCGACGCCACCAACTTCTCCCTGCTGAAGCAGGCCGGCGACGTGGAGGAGAACCCCGGCCCCGGATCCATGGACTACAAAGACCATGACGGTGATTATAAAGATCATGACATCGATTACAAGGATGACGATGACAAGGGTAGTGGGAGCGGTTCCAAAGGAGAAGAACTGTTTACCGGTGTTGTGCCAATTTTGGTTGAACTCGATGGTGATGTCAACGGACATAAGTTCTCAGTGAGAGGCGAAGGAGAAGGTGACGCCACCATTGGAAAATTGACTCTTAAATTCATCTGTACTACTGGTAAACTTCCTGTACCATGGCCGACTCTCGTAACAACGCTTACGTACGGAGTTCAGTGCTTTTCGAGATACCCAGACCATATGAAAAGACATGACTTTTTTAAGTCGGCTATGCCTGAAGGTTACGTGCAAGAAAGAACAATTTCGTTCAAAGATGATGGAAAATATAAAACTAGAGCAGTTGTTAAATTTGAAGGAGATACTTTGGTTAACCGCATTGAACTGAAAGGAACAGATTTTAAAGAAGATGGTAATATTCTTGGACACAAACTCGAATACAATTTTAATAGTCATAACGTATACATCACTGCTGATAAGCAAAAGAACGGAATTAAAGCGAATTTCACAGTACGCCATAATGTAGAAGATGGCAGTGTTCAACTTGCCGACCATTACCAACAAAACACCCCTATTGGAGACGGTCCGGTACTTCTTCCTGATAATCACTACCTCTCAACACAAACAGTCCTGAGCAAAGATCCAAATGAAAAATCTAGAGGTAGTGGGAGCGGTATCACCACCGTGGAGTCCAACTCCTCCTGGTGGACCAACTGGGTGATCCCCGCCATCTCCGCCCTGGTGGTGGCCCTGATGTACCGCCTGTACATGGCCGAGGACTAGGCGGCCGCGGATCTTTGTGAAGG | IDT | N/A |
