# Supplementary material for: Cholesterol transfer proteins promote Atg-independent ER clearance by lysosomes
Source: Cell Rep. Author manuscript; Available in PMC 2026 Jul 13. (PMC13358760; doi:10.1016/j.celrep.2026.117537)
Supplement: 3 [file NIHMS2190755-supplement-3.docx]

**SUPPLEMENTAL TABLE 2. Genotypes related to each figure.**

| ***Drosophila* genotypes related to each figure.** | |
| --- | --- |
| **Figure** | **Genotype** |
| **Figure 1** |  |
| A | *w*, Atg8a^Δ^/w*,+;+;+* |
| B | *w*, Atg8a^Δ^/w*, Atg8a^Δ^;+;+* |
| C | *y*, w*;*+;*Atg16^Δ^/Atg16^Δ^* |
| D | y*, w*, hs::Flp/w* (or y); FRT42D, Ubi::RFP/FRT42D, *Vps25^A3^*; Sec61α-GFP/+ |
| E | y*, w*, hs::Flp/w* (or y); FRT42D, Ubi::RFP/FRT42D, *Atg9^D51^*; Sec61α-GFP/+ |
| F | y*, w*, hs::Flp/w* (or y); FRT42D, Ubi::RFP/FRT42D, *Atg9^D51^, Vps25^A3^*; Sec61α-GFP/+ |
| **Figure 2** |  |
| A | y*, w*, hs::Flp, Ubi::RFP, FRT19A/*Vap33^Δ448^*, FRT19A;+;Sec61α-GFP/+ |
| B | y*, w*, hs::Flp, Ubi::RFP, FRT19A/*Atg8a^Δ^*, FRT19A;+;Sec61α-GFP/+ |
| C | y*, w*, hs::Flp, Ubi::RFP, FRT19A/*Vap33^Δ448^, Atg8a^Δ^,* FRT19A;+;Sec61α-GFP/+ |
| E | *w**, *Atg8a^Δ^/w*,+*; NP1::GAL4/*Vap33^TKO^*; UAS::Cas9/+ |
| F | *w**, *Atg8a^Δ^/y*; NP1::GAL4/*+*; UAS::Cas9/+ |
| G | *w**, *Atg8a^Δ^/y*; NP1::GAL4/*Vap33^TKO^*; UAS::Cas9/+ |
| I | y*, w*, hs::Flp, V5-3×FLAG-Vap33/w* (or y);FRT42D/FRT42D, *Atg9^D51^* |
| **Figure 3** |  |
| B | y*, w*, hs::Flp/w* (or y);FRT42D/FRT42D, *Atg9^D51^*;V5-3×FLAG-Osbp/+ |
| E | y*, w*, hs::Flp/w* (or y);+;FRT82B, Ubi::RFP/Sec61α-GFP, FRT82B, *Osbp^Δ^* |
| F | y*, w*, hs::Flp/w* (or y);+;FRT82B, Ubi::RFP/Sec61α-GFP, FRT82B, *Atg16^Δ^* |
| G | y*, w*, hs::Flp/w* (or y);+;FRT82B, Ubi::RFP/Sec61α-GFP, FRT82B, *Atg16^Δ^*, *Osbp^Δ^* |
| I | y*, w*, hs::Flp, Ubi::RFP, FRT19A/*Vap33^Δ448^*, FRT19A;+;Sec61α-GFP/+ |
| J | y*, w*, hs::Flp/w* (or y);+;FRT82B, Ubi::RFP/Sec61α-GFP, FRT82B, *Osbp^Δ^* |
| K | y*, w*, hs::Flp, Ubi::RFP, FRT19A/*Vap33^Δ448^*, FRT19A;+;FRT82B, Ubi::RFP/Sec61α-GFP, FRT82B, *Osbp^Δ^* |
| M | y*, w*, hs::Flp/w* (or y);FRT42D, Ubi::RFP/FRT42D, *Start1^Δ^*;Sec61α-GFP/+ |
| N | y*, w*, hs::Flp/w* (or y);FRT42D, Ubi::RFP/FRT42D, *Atg9^D51^*;Sec61α-GFP/+ |
| O | y*, w*, hs::Flp/w* (or y);FRT42D, Ubi::RFP/FRT42D, *Atg9^D51^, Start1^Δ^*;Sec61α-GFP/+ |
| Q | y*, w*, hs::Flp/w* (or y);+;FRT82B, Ubi::RFP/Sec61α-GFP, FRT82B, *Osbp^Δ^* |
| R | y*, w*, hs::Flp/w* (or y);FRT42D, Ubi::RFP/FRT42D, *Start1^Δ^*;Sec61α-GFP/+ |
| S | y*, w*, hs::Flp/w* (or y);FRT42D, Ubi::RFP/FRT42D, *Start1^Δ^*;FRT82B, Ubi::RFP/Sec61α-GFP, FRT82B, *Osbp^Δ^* |
| **Figure 4** |  |
| C | y*, w*, hs::Flp/w* (or y); NP1::GAL4, FRT42D, Ubi::RFP/FRT42D, *Atg9^D51^*; Tub::Gal80^ts^/UAS::Lyso-G11-G(1-10)-ER |
| D | y*, w*, hs::Flp/w* (or y); NP1::GAL4, FRT42D, Ubi::RFP/FRT42D, *Atg9(D51), Start1Δ*; Tub::Gal80^ts^/UAS::Lyso-G11-G(1-10)-ER |
| F | y*, w*, hs::Flp/w* (or y); FRT42D/FRT42D, *Atg9^D51^*; V5-3×FLAG-Osbp/+ |
| G | y*, w*, hs::Flp/w* (or y); FRT42D/FRT42D, *Atg9^D51^, Start1^Δ^*; V5-3×FLAG-Osbp/+ |
| **Figure 5** |  |
| B | y*, w*, hs::Flp/w* (or y);FRT42D, Ubi::RFP/FRT42D, *Atg9^D51^*; Sec61α-GFP/+ |
| C | y*, w*, hs::Flp/w* (or y);FRT42D, Ubi::RFP/FRT42D,  *Start1^ΔFFAT^*;Sec61α-GFP/+ |
| D | y*, w*, hs::Flp/w* (or y);FRT42D, Ubi::RFP/FRT42D,  *Start1^ΔChol^*;Sec61α-GFP/+ |
| E | y*, w*, hs::Flp/w* (or y);FRT42D, Ubi::RFP/FRT42D, *Atg9^D51^, Start1^ΔFFAT^*;Sec61α-GFP/+ |
| F | y*, w*, hs::Flp/w* (or y);FRT42D, Ubi::RFP/FRT42D, *Atg9^D51^, Start1^ΔChol^*;Sec61α-GFP/+ |
| **Figure 6** |  |
| C | y*, w*, hs::Flp/w* (or y);NP1::GAL4, FRT42D, Ubi::RFP/FRT42D, *Atg9^D51^*;Tub::Gal80^ts^/UAS::D4H-G11-G(1-10)-ER |
| D | y*, w*, hs::Flp/w* (or y);NP1::GAL4, FRT42D, Ubi::RFP/FRT42D, *Atg9^D51^, Start1Δ*;Tub::Gal80^ts^/UAS::D4H-G11-G(1-10)-ER |
| E | y*, w*, hs::Flp/w* (or y);NP1::GAL4, FRT42D, Ubi::RFP/FRT42D, *Atg9^D51^, Start1^ΔFFAT^*;Tub::Gal80^ts^/UAS::D4H-G11-G(1-10)-ER |
| F | y*, w*, hs::Flp/w* (or y);NP1::GAL4, FRT42D, Ubi::RFP/FRT42D, *Atg9^D51^, Start1^ΔChol^*;Tub::Gal80^ts^/UAS::D4H-G11-G(1-10)-ER |
| **Figure 7** |  |
| A | y*, w*, hs::Flp/w* (or y);FRT42D, Ubi::RFP/FRT42D, *Atg9^D51^*;+ |
| B | y*, w*, hs::Flp/w* (or y);FRT42D, Ubi::RFP/FRT42D, *Atg9^D51^, Start1^Δ^*;+ |
| C | y*, w*, hs::Flp/w* (or y);FRT42D, Ubi::RFP/FRT42D, *Atg9^D51^, Start1^ΔFFAT^*;+ |
| D | y*, w*, hs::Flp/w* (or y);FRT42D, Ubi::RFP/FRT42D, *Atg9^D51^, Start1^ΔChol^*;+ |
| **Figure S1** |  |
| A | y*, w*; NP1::Gal4/+; UAS::*luciferase* RNAi/+ |
| B | y*, w*; NP1::Gal4/+; UAS::*Vps25* RNAi/+ |
| D and E | y*, w*, hs::Flp, His2Av::GFP, FRT19A/*Atg8a^Δ^*, FRT19A; FRT42D, Ubi::RFP/FRT42D, *Atg9^D51^* |
| H | y*, w*, hs::Flp/w* (or y); NP1::Gal4/+;FRT82B/UAS::ss-pHluorin-mKate2-KDEL-V5, FRT82B, *Lsn^ΔSS6^* |
| I | y*, w*, hs::Flp/w* (or y); NP1::Gal4/+;FRT82B/UAS::ss-pHluorin-mKate2-KDEL-V5, FRT82B, *Atg16^Δ^* |
| J | y*, w*, hs::Flp/w* (or y); NP1::Gal4/+;FRT82B/UAS::ss-pHluorin-mKate2-KDEL-V5, FRT82B, *Lsn^ΔSS6^,Atg16^Δ^* |
| **Figure S2** |  |
| A | y*, w*, FRT19A/*Atg8^Δ^*, FRT19A; NP1::GAL4, UAS::ss-pHluorin-mKate2-KDEL-V5/+;hs::Flp/+ |
| B | y*, w*, FRT19A/*Vap33^Δ448^*, *Atg8^Δ^*, FRT19A; NP1::GAL4, UAS::ss-pHluorin-mKate2-KDEL-V5/+; hs::FlpD5/+ |
| D | y*, w*, hs::Flp, His2Av::GFP, FRT19A/*Vap33^Δ448^*, FRT19A;+;+ |
| E | y*, w*, hs::Flp, His2Av::GFP, FRT19A/ *Atg8a^Δ^*, FRT19A;+;+ |
| F | y*, w*, hs::Flp, His2Av::GFP, FRT19A/*Vap33^Δ448^, Atg8a^Δ^,* FRT19A;+;+ |
| H | y*, w*, hs::Flp, His2Av::GFP, FRT19A/*Vap33^Δ448^*, FRT19A;+;+ |
| K | y*, w*, hs::Flp, His2Av::GFP, FRT19A/*Vap33^Δ448^*, FRT19A;+;+ |
| M | y*, w*, hs::Flp, His2Av::GFP, FRT19A/*Vap33^Δ448^*, FRT19A;+; V5-3×FLAG-Osbp/+ |
| **Figure S3** |  |
| C | y*, w*, hs::Flp/w* (or y); NP1::GAL4, UAS::ss-pHluorin-mKate2-KDEL-V5/+; FRT82B, Ubi::RFP/FRT82B, *Atg16^Δ^* |
| D | y*, w*, hs::Flp/w* (or y); NP1::GAL4, UAS::ss-pHluorin-mKate2-KDEL-V5/+; FRT82B, Ubi::RFP/FRT82B, *Atg16^Δ^*, *Osbp^Δ^* |
| F | y*, w*, hs::Flp/w* (or y);+;FRT82B, Ubi::GFP/FRT82B, *Osbp^Δ^* |
| I | y*, w*, hs::Flp/w* (or y);+;FRT82B, Ubi::GFP/FRT82B, *Osbp^Δ^* |
| **Figure S4** |  |
| B | y*, w*, hs::Flp/w* (or y); NP1::GAL4, FRT42D, Ubi::RFP/FRT42D, *Atg9^D51^*; UAS::ss-pHluorin-mKate2-KDEL-V5/+ |
| C | y*, w*, hs::Flp/w* (or y); NP1::GAL4, FRT42D, Ubi::RFP/FRT42D, *Atg9^D51^, Start1^Δ^*; UAS::ss-pHluorin-mKate2-KDEL-V5/+ |
| **Figure S5** |  |
| A | y*, w*, hs::Flp/w* (or y); FRT42D, Ubi::RFP/FRT42D, *Atg9^D51^*;+ |
| B | y*, w*, hs::Flp/w* (or y); FRT42D, Ubi::RFP/FRT42D, *Atg9^D51^, Start1^Δ^*;+ |
| C | y*, w*, hs::Flp/w* (or y); FRT42D, Ubi::RFP/FRT42D, *Atg9^D51^, Start1^ΔFFAT^*;+ |
| D | y*, w*, hs::Flp/w* (or y); FRT42D, Ubi::RFP/FRT42D, *Atg9^D51^, Start1^ΔChol^*;+ |
| F | y*, w*, hs::Flp/w* (or y); NP1::GAL4, FRT42D, Ubi::RFP/FRT42D, *Atg9^D51^*; Tub::Gal80ts/UAS::Lyso-G11-G(1-10)-ER |
| G | y*, w*, hs::Flp/w* (or y); NP1::GAL4, FRT42D, Ubi::RFP/FRT42D, *Atg9^D51^, Start1^Δ^*; Tub::Gal80^ts^/UAS::Lyso-G11-G(1-10)-ER |
| H | y*, w*, hs::Flp/w* (or y); NP1::GAL4, FRT42D, Ubi::RFP/FRT42D, *Atg9^D51^, Start1^ΔFFAT^*; Tub::Gal80^ts^/UAS::Lyso-G11-G(1-10)-ER |
| I | y*, w*, hs::Flp/w* (or y); NP1::GAL4, FRT42D, Ubi::RFP/FRT42D, *Atg9^D51^, Start1^ΔChol^*; Tub::Gal80^ts^/UAS::Lyso-G11-G(1-10)-ER |
| **Figure S6** |  |
| A | y*, w*, hs::Flp/w* (or y); Ubi::GFP, FRT40A/*Npc1a^Δ^*, FRT40A;+ |
| C | y*, w*, hs::Flp/w* (or y); Ubi::RFP, FRT40A/*Npc1a^Δ^*, FRT40A;V5-3×FLAG-Osbp/+ |
| E | y*, w*, hs::Flp/w* (or y); Ubi::RFP, FRT40A/*Npc1a^Δ^*, FRT40A;Sec61α-GFP/+ |
